# Early cost-utility analysis of tissue-engineered heart valves compared to bioprostheses in the aortic position in elderly patients

**DOI:** 10.1007/s10198-020-01159-y

**Published:** 2020-01-25

**Authors:** Simone A. Huygens, Isaac Corro Ramos, Carlijn V. C. Bouten, Jolanda Kluin, Shih Ting Chiu, Gary L. Grunkemeier, Johanna J. M. Takkenberg, Maureen P. M. H. Rutten-van Mölken

**Affiliations:** 1 Department of Cardiothoracic Surgery, Erasmus MC, University Medical Center, Rotterdam, The Netherlands; 2 Erasmus School of Health Policy and Management, Erasmus University, Rotterdam, The Netherlands; 3 Institute for Medical Technology Assessment, Erasmus University, Rotterdam, The Netherlands; 4 Department of Biomedical Engineering, Eindhoven University of Technology, Eindhoven, The Netherlands; 5 Department of Cardio-Thoracic Surgery, Academic Medical Centre, Amsterdam, The Netherlands; 6 Medical Data Research Centre, Providence Health and Service, Portland, OR USA

**Keywords:** Early health technology assessment, Patient-level simulation model, Heart valve implantation, Tissue-engineered heart valves, I18, I19

## Abstract

**Objectives:**

Aortic valve disease is the most frequent indication for heart valve replacement with the highest prevalence in elderly. Tissue-engineered heart valves (TEHV) are foreseen to have important advantages over currently used bioprosthetic heart valve substitutes, most importantly reducing valve degeneration with subsequent reduction of re-intervention. We performed early Health Technology Assessment of hypothetical TEHV in elderly patients (≥ 70 years) requiring surgical (SAVR) or transcatheter aortic valve implantation (TAVI) to assess the potential of TEHV and to inform future development decisions.

**Methods:**

Using a patient-level simulation model, the potential cost-effectiveness of TEHV compared with bioprostheses was predicted from a societal perspective. Anticipated, but currently hypothetical improvements in performance of TEHV, divided in durability, thrombogenicity, and infection resistance, were explored in scenario analyses to estimate quality-adjusted life-year (QALY) gain, cost reduction, headroom, and budget impact.

**Results:**

Durability of TEHV had the highest impact on QALY gain and costs, followed by infection resistance. Improved TEHV performance (− 50% prosthetic valve-related events) resulted in lifetime QALY gains of 0.131 and 0.043, lifetime cost reductions of €639 and €368, translating to headrooms of €3255 and €2498 per hypothetical TEHV compared to SAVR and TAVI, respectively. National savings in the first decade after implementation varied between €2.8 and €11.2 million (SAVR) and €3.2–€12.8 million (TAVI) for TEHV substitution rates of 25–100%.

**Conclusions:**

Despite the relatively short life expectancy of elderly patients undergoing SAVR/TAVI, hypothetical TEHV are predicted to be cost-effective compared to bioprostheses, commercially viable and result in national cost savings when biomedical engineers succeed in realising improved durability and/or infection resistance of TEHV.

**Electronic supplementary material:**

The online version of this article (10.1007/s10198-020-01159-y) contains supplementary material, which is available to authorized users.

## Introduction

Aortic valve disease is the most frequent indication for heart valve surgery [1]. Prevalence of aortic valve disease is the highest in elderly patients (stenosis 2.8%; regurgitation 2.0%), due to degeneration of the native aortic valve [2]. Aortic valve disease can be treated with medication to relieve symptoms, but can only be cured with aortic valve replacement [3]. The average annual number of patients undergoing surgical aortic valve replacement (SAVR) in 2007–2015 in The Netherlands was 1931 (Adult Cardiac Surgery Database [ACSD]). In addition to SAVR, transcatheter aortic valve implantation (TAVI) is a less invasive alternative to replace the aortic valve for patients who are deemed inoperable or at high operable risk because of comorbidities [3]. In 2013, 809 patients underwent TAVI in The Netherlands and this number is expected to increase to approximately 3745 patients per year [4, 5]. Due to the ageing population and improvements in healthcare, the number of aortic valve implantations is only expected to increase further, especially in elderly patients [2, 6].

During a TAVI procedure, a balloon or self-expanding bioprosthesis is implanted with a catheter through an artery most frequently in the groin or underneath the collarbone. Surgical heart valve substitutes can be divided into biological (human or animal donor) and mechanical valves. In elderly patients eligible for surgery, bioprostheses (animal donor) are preferred because patients’ life expectancy is usually shorter than the valve’s durability and therefore, patients can benefit from the advantages of bioprostheses (e.g., no need for lifelong anticoagulation) [3]. However, risk of re-intervention due to limited durability of bioprostheses is not absent in elderly patients and there is an increased risk of endocarditis (i.e. infected heart valve) after SAVR and TAVI [7, 8].

Tissue-engineered heart valves (TEHV) can potentially limit the disadvantages of existing heart valve substitutes [6, 9–11]. Lately, emphasis has shifted towards the development of in situ TEHV [10]. In this approach, valve-shaped scaffolds are implanted in the heart that recruit cells from the bloodstream and surrounding tissues and gradually transform into a living valve, while the scaffold degrades [11]. Ideally, TEHV would remodel themselves and last a lifetime in the same way as most native heart valves do. Currently, both surgical and transcatheter implantations of in situ TEHV are being explored. Preclinical studies on TEHV performance in sheep and clinical trials of tissue-engineered vascular grafts in humans showed promising results, but results of a first-in-man clinical trial are not available yet [9–12].

As TEHV are still under development, biomedical engineers requested guidance on which aspects of heart valve performance they should focus to improve clinical outcomes and achieve cost-effectiveness. Further, they wanted to know whether elderly patients in need of aortic valve replacement would be among the target populations. To guide the further development of TEHV, we performed an early (sometimes referred to as prospective) Health Technology Assessment (HTA) study to predict the potential cost-effectiveness, headroom and budget impact of hypothetical TEHV compared with bioprostheses in elderly patients requiring surgical or transcatheter aortic valve implantation using a patient-level simulation model.

## Methods

The methods and reporting of this study conform to Consolidated Health Economic Evaluation Reporting Standards (CHEERS, Supplementary material) [13].

### Study population

The study population that is simulated was sampled with replacement from existing patient databases and comprised patients of ≥ 70 years who had an aortic bioprosthetic valve implantation, either through SAVR or TAVI. SAVR patients were sampled from the ACSD from The Netherlands Association for Cardio-Thoracic Surgery (n = 15,405, mean ± SD age = 77.0 ± 4.1 years). TAVI patients were sampled from Dutch health insurance claims databases (n = 809, mean ± SD age = 81.9 ± 4.9 years) [4]. Supplement 1 provides more information on the databases and presents patient and intervention characteristics of the study populations.

### Patient-level simulation model

**Fig. 1:**
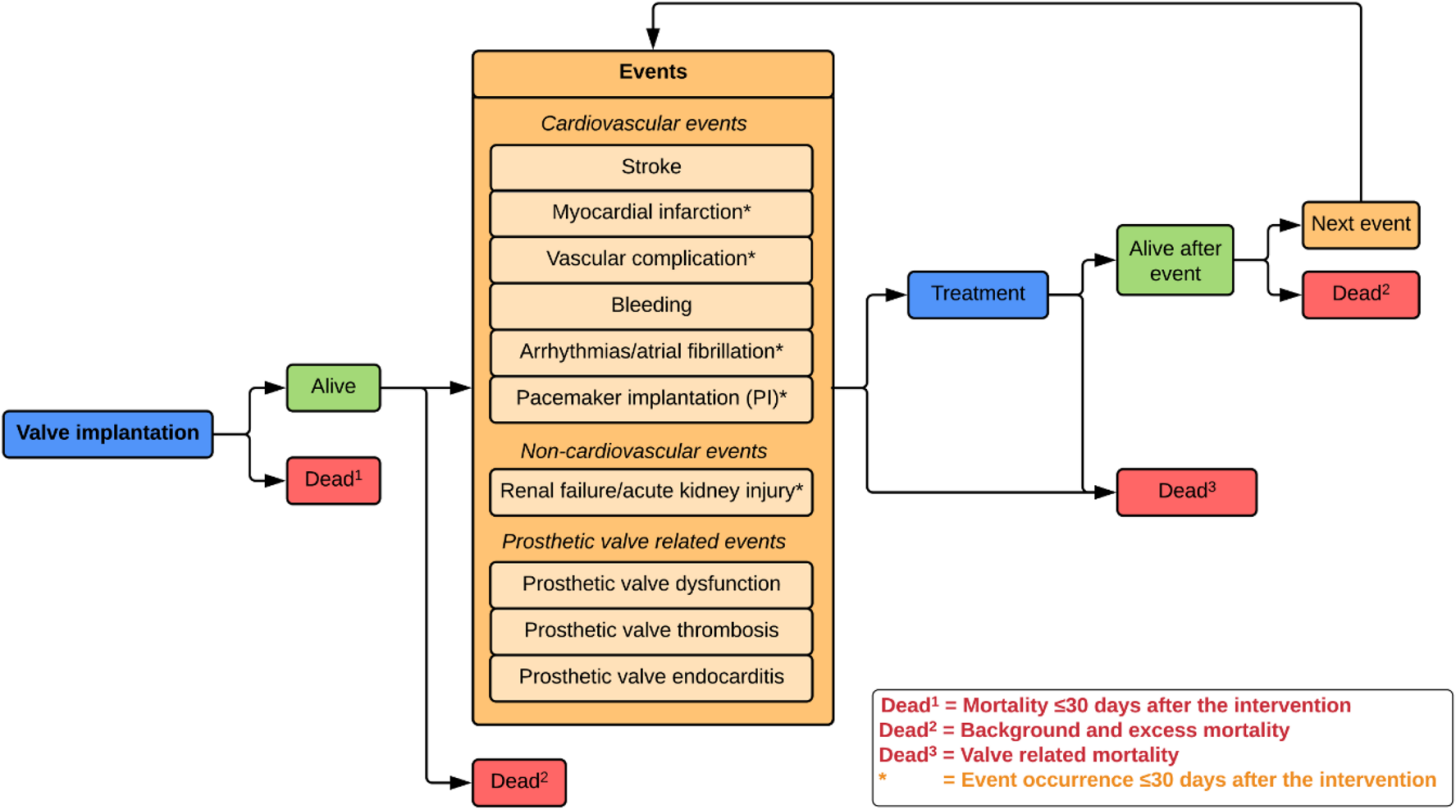
Conceptual model. Adaptations of the original conceptual model are discussed in Supplement 2

We chose a patient-level simulation model (more specifically a discrete event simulation (DES) model) over a decision tree or cohort state transition model (also known as Markov model) because it has the ability to incorporate recurrence of events and to “remember patient history” without leading to an unmanageable number of health states [14, 15]. The patient-level simulation model was based on a published conceptual model developed previously (Fig. 1/Supplement 2) [16]. Figure S1 illustrates the flowchart of the patient-level simulation model and Table S2 provides an overview of the input parameters. The model combines fixed estimates and regression equations for different intermediate and final outcomes (Table S3).

The model simulation starts with randomly sampling 25,000 patients from the databases specified above. The number of 25,000 sampled patients was required to get stable results. For each patient, the model starts with the valve implantation. The period after the valve implantation until death is divided into an early (≤ 30 days after the intervention) and late period (> 30 days after the intervention) to be in line with guidelines for reporting outcomes after cardiac valve interventions [17]. The following events are included in our model during the entire simulation (i.e. both as early and late events): stroke, bleeding, prosthetic valve dysfunction (structural valve deterioration (SVD) and non-structural valve dysfunction; including calcification, structural and residual leak, and thickening of the prosthetic valve), -thrombosis and -endocarditis. In addition, the following events are only included within 30 days after the intervention (i.e. early events) as it was not expected that long-term occurrence would be related to the heart valve intervention: myocardial infarction, vascular complication, arrhythmias/atrial fibrillation, pacemaker implantation, renal failure/acute kidney injury. Patients can experience multiple early events within 30 days after the intervention. Subsequently, time to late events (i.e. stroke, bleeding, prosthetic valve dysfunction, -thrombosis and -endocarditis) and death are calculated (independent of patient and intervention characteristics or patient history). Late mortality is divided into mortality directly related to valve-related events, background mortality, and excess mortality. Excess mortality is the mortality ascribed to the potential excess risk of dying of patients after heart valve interventions. The event (including death) with the lowest predicted time value is considered to occur after which the consequences for costs and quality-adjusted life years (QALY) are modelled. Then, times to late events and death are recalculated. The simulation stops when death has the lowest predicted time value of all events or when patients die directly after an event. This process is repeated for all patients. By combining data of all simulated patients, the average difference in QALYs and costs between TEHV and bioprostheses is calculated. The model was implemented in R 3.3.2 using RStudio 1.0.136.

### Model input and assumptions

#### Mortality and events

Mortality was divided into early mortality (≤ 30 days), mortality directly related to valve-related events, background mortality, and excess mortality. Background mortality was obtained for the year 2016 in the Dutch general population [18]. Excess mortality was expressed as hazard ratio relative to background mortality (SAVR: 0.86 [8]; TAVI: 1.50 (Supplement 3)). This means that background mortality in SAVR patients was 14% lower than in the general population, probably due to careful selection of relatively healthy elderly to undergo SAVR while frail elderly are rejected for surgery [8, 19]. Background mortality in TAVI patients was 50% higher than in the general population, possibly due to increased occurrence of comorbidities in TAVI patients [20].

Clinical input parameters are provided in Table 1. Risks of early mortality, stroke, renal failure, arrhythmias and myocardial infarction within 30 days after SAVR were dependent on patient and intervention characteristics, estimated using logistic regression models based on ACSD (Supplement 4). All other mortality and events risks and rates were independent of patient and intervention characteristics or patient history. The occurrence of late events after SAVR was based on our previously performed systematic review and meta-analysis [8]. The clinical outcomes after TAVI were derived from a systematic review performed by Gargiulo et al. [21] (Supplement 4). Risks and rates of other early events after SAVR and probabilities of re-intervention or death as a direct result of events were derived from literature (references in Table 1). Time to SVD after SAVR is obtained from a Gompertz distribution fitted to a pooled Kaplan–Meier curve [8]. Time to SVD after TAVI is obtained from a lognormal distribution fitted to a published Kaplan–Meier curve [7]. These distributions had the best fit according to visual comparison, log-likelihood and Akaike information criterion (Table S12, Figures S2–15). We were unable to determine distributions of other events due to limited data availability; therefore, we assumed constant hazard rates using exponential distributions.

**Table 1:** Clinical input parameters

	SAVR	Distribution	Source	TAVI	Distribution	Source
*Early mortality (%)*						
After initial intervention	3.9*	Multivariate normal^7^	ACSD	5.4	Beta (α 65, β 1135)	[21]
After re-intervention	9.0*	Multivariate normal^7^	ACSD	8.6^3^	Uniform (± 10%)	[41]
*Early events (%)*						
Stroke	2.5*	Multivariate normal^7^	ACSD	2.9	Beta (α 58, β 1919)	[21]
Myocardial infarction	1.6*	Multivariate normal^7^	ACSD	1.0	Beta (α 20, β 1983)	[21]
Vascular complications	–	–		8.1	Beta (α 50, β 565)	[21]
Bleeding^1^	4.2	Beta (α 77, β 1761)	[8]	8.7	Beta (α 11, β 115)	[21]
Arrhythmias/atrial fibrillation	41.5*	Multivariate normal^7^	ACSD	11.0	Beta (α 31, β 249)	[21]
Pacemaker implantation (PI)	8.1	Beta (α 4, β 48)	[8]	12.2	Beta (α 85, β 610)	[21]
Renal failure/acute kidney injury	3.4*	Multivariate normal^7^	ACSD	4.5	Beta (α 10, β 215)	[21]
Prosthetic valve dysfunction^2^	–	–	Assumption	6.8	Beta (α 30, β 405)	[21]
Prosthetic valve thrombosis	–	–	Assumption	–	–	Assumption
Prosthetic valve endocarditis	–	–	Assumption	–	–	Assumption
*Late events (%/year ± SD)*						
Stroke	0.77 ± 0.28	Lognormal	[8]	0.96 ± 0.10^4^	Lognormal	[8, 42]
Probability of dying (%)	44.0	Beta (α 11, β 14)	[8]	44.0	Beta (α 11, β 14)	[8]
Bleeding	0.75 ± 0.16	Lognormal	[8]	0.95 ± 0.35^4^	Lognormal	[8, 42]
Probability of dying (%)	39.1	Beta (α 18, β 28)	[8]	39.1	Beta (α 18, β 28)	[8]
Structural valve deterioration	Rate: 0.003 ± 0.001; Shape: 0.124 ± 0.024	Gompertz	[8]	Lognormal; mean log 2.711 ± 0.379; SD log 0.613 ± 0.335	Lognormal	[7]
Probability of dying (%)	17.0	Dirichlet^6^ (α^1^ 18, α^2^ 45, α^3^ 41)	[43]	17.0	Dirichlet^6^ (α^1^ 18, α^2^ 45, α^3^ 41)	[43]
Probability of re-intervention (%)	43.3		[8]	25.0		[7]
Probability TAVI	6.2	Uniform (6.1–6.3)	[5]	100	Assumption	
Probability SAVR	93.8		[5]	0	Assumption	
Probability conservative treatment	39.7			58.0	Assumption	
Probability TAVI	61.7	Uniform (42.0–81.7)	[5]	0	Assumption	
Probability medical treatment	38.3		[5]	100	Assumption	
Nonstructural valve dysfunction	0.47 ± 0.27	Lognormal	[8]	–	Assumption	
Probability of dying (%)	5.0	Dirichlet^6^ (α^1^ 1, α^2^ 10, α^3^ 15)	[43]	–	–	
Probability of re-intervention (%)	38.5		[8]	–		
Prosthetic valve thrombosis	0.12 ± 0.09	Lognormal	[8]	0.24^5^	Uniform (± 20%)	[44]
Probability of dying (%)	0.0	Dirichlet^6^ (α^1^ 0, α^2^ 2, α^3^ 15)	[43]	0.0	Dirichlet^6^ (α^1^ 0, α^2^ 3, α^3^ 23)	[43]
Probability of re-intervention (%)	0.12		[45]	0.12		[46]
Prosthetic valve endocarditis	0.57 ± 0.08	Lognormal	[8]	0.54 ± 0.10	Lognormal	[21]
Probability of dying (%)	34.0	Dirichlet^6^ (α^1^ 26, α^2^ 37, α^3^ 13)	[43]	34.0	Dirichlet^6^ (α^1^ 26, α^2^ 37, α^3^ 13)	[43]
Probability of re-intervention (%)	49.0		[8]	49.0		[8]
Hazard ratio excess mortality	0.86	Uniform (± 10%)	[8]	1.40	Uniform (± 10%)	This study

*Mean (95% CI) in the Adult Cardiac Surgery Database (ACSD). Risk in the patient-level simulation model dependent on patient and intervention characteristics using logistic regression formula. “-“Not reported in any of the studies, therefore assumed not to occur

^1^Definition of bleeding is reexploration for bleeding after SAVR and major bleedings after TAVI

^2^Paravalvular leak after TAVI

^3^Hazard ratio of 1.6 applied to early mortality risk of initial intervention

^4^Hazard ratio of SAVR patients compared to the general population applied to occurrence in age and sex matched general population for the TAVI population

^5^Blackstone & Kirklin have shown that valve thrombosis mainly occurs during the first year after surgical mechanical aortic valve implantation and deteriorates to almost zero after six years [47]. The higher occurrence in the early phase may be caused by suboptimal anticoagulation treatment in the first post-intervention period. Since, the mean follow-up of the Bern TAVI Registry was only one year, it is likely that the occurrence rate of valve thrombosis after TAVI found in this study will not remain constant but will reduce over time. Therefore, we recalculated the linearized occurrence rate of 0.69%/patient-year, assuming that it will be zero from year 7 onwards

^6^Dirichlet distribution parameters: α^1^ = number of deaths, α^2^ = number of re-interventions, α^3^ = number of other treatment

^7^Multivariate normal distribution: coefficients of the regression model are randomly drawn from a multivariate normal distribution based on coefficients and variance–covariance matrix

#### Costs

The majority of the healthcare cost inputs were derived from our previously performed retrospective cost-analysis of Dutch health insurance claims data [4]. Healthcare costs were divided into intervention (procedure and hospital stay), event, other healthcare (healthcare use not directly related to the heart valve intervention or initial treatment of associated events), and end-of-life healthcare (healthcare use associated with dying) costs (Table 2). Healthcare costs were defined as expenditures reimbursed by health insurers. Costs were dependent on patient and intervention characteristics using (multilevel) generalised linear models ((M)GLM) (Table S12/ [4]), except for costs of bleeding and conservative treatment of prosthetic valve-related events. We assumed most events had a permanent influence on healthcare use (e.g., lifelong follow-up with cardiologist after pacemaker implantation). Hence, other healthcare costs were assumed to be increased for the remaining patient’s lifetime after most events, except for prosthetic valve-related events and re-intervention to avoid double counting of follow-up costs for the initial heart valve implantation. Other healthcare costs were estimated with the MGLM regression formula within three years after the intervention (Table S9). Beyond three years, these costs were adjusted to patient age using relative increases in total healthcare costs by age and sex of the Dutch general population [22].

**Table 2:** Costs and utilities

			Distribution	Source
*Intervention costs*				
SAVR	25,474		Multivariate normal^4^	[4]*
TAVI	33,178		Multivariate normal^4^	[4]*
*Event treatment costs*				
Stroke	3054		Multivariate normal^4^	[4]*
Myocardial infarction	5157		Multivariate normal^4^	[4]*
Vascular complications	5112		Uniform (∓ 20%)	[48]
Reexploration for bleeding	5048		Uniform (∓ 20%)	[48]
Bleeding	1617		Uniform (∓ 20%)	[48]
Atrial fibrillation (without PI)	1225		Multivariate normal^4^	[4]*
Pacemaker implantation (PI)	11,738		Multivariate normal^4^	[4]*
Acute kidney injury/renal failure	9650		Multivariate normal^4^	[4]*
Prosthetic valve dysfunction	1478		Uniform (∓ 20%)	[49–54]
Prosthetic valve thrombosis	5824		Uniform (∓ 20%)	[53–55]
Prosthetic valve endocarditis	8923		Multivariate normal^4^	[4]*
Re-intervention SAVR	25,936		Multivariate normal^4^	[4]*
Re-intervention TAVI	33,178		Multivariate normal^4^	[4]*
*Other healthcare cost*^*1*^				
Post-intervention year 1	18,479		Multivariate normal^4^	[4]*
Post-intervention year 2	10,607			[4]*
Post-intervention year 3	10,832			[4]*
*Productivity costs of unpaid work*^*3*^	*Costs per month*			
SAVR	44		Multivariate normal^4^	[23]
TAVI	50		Multivariate normal^4^	[23]
*Informal care costs*^*3*^	*Costs per month*			
SAVR	164		Multivariate normal^4^	[23]
TAVI	388		Multivariate normal^4^	[23]
*Utilities at start of the simulation*				
SAVR	0.837		Multivariate normal^4^	[23]
TAVI	0.718		Multivariate normal^4^	[23]
*Utilities after events*	*Utility multiplier*	*Duration*		
Stroke	0.841	Lifetime	Uniform^5^	[56, 57]
Myocardial infarction	0.914	1 year	Uniform^5^	[58, 59]
Vascular complications	0.981	1 week	Uniform^5^	[60]
Bleeding	0.965	1 year	Uniform^5^	[61]
Atrial fibrillation (without PI)	0.955	1 year	Uniform^5^	[62]
Pacemaker implantation (PI)	0.804	1 month	Uniform^5^	[63]
Acute kidney injury/renal failure	0.804	1 year	Uniform^5^	[64]
Re-intervention	0.946	SAVR/TAVI: 4/1 month(s)	Uniform^5^	[65]/[8]
Conservative treatment of:				
Prosthetic valve dysfunction	0.886^2^	Lifetime	Uniform^5^	[50, 66]
Prosthetic valve thrombosis	0.968^2^	10 days	Uniform^5^	[55, 67]
Prosthetic valve endocarditis	0.968^2^	6 weeks	Uniform^5^	[67, 68]

*Mean in the Vektis database adjusted to 2016€. Costs in the model dependent on patient and intervention characteristics using (M)GLM [4]

^1^Mean total healthcare costs per year including costs of treatment of events and death (costs types are estimated separately in the model), but excluding intervention costs. Costs are based on data of SAVR patients, but it is assumed they are also applicable to TAVI patients

^2^Conservative treatment, no re-intervention

^3^Mean across all patients, including patients without unpaid work or informal care

^4^Multivariate normal distribution: coefficients of the regression model are randomly drawn from a multivariate normal distribution based on coefficients and variance–covariance matrix

^5^50% deviation of 1-utility multiplier to prevent the utility multiplier from exceeding 1

Costs beyond healthcare included productivity costs of unpaid work and informal care costs and were based on results from a patient-reported questionnaire published previously [23]. These costs were dependent on patient and intervention characteristics based on logistic models and GLM (Tables S13–14). Productivity costs of paid work were excluded because the vast majority (≥ 95%) of elderly patients undergoing SAVR or TAVI do not have paid employment [23]. Productivity costs were increased after events by assuming that patients were unable to perform their unpaid work activities during hospital admissions for bleeding and prosthetic valve-related events (Table S15), 4 months after surgical re-intervention [23], 1 month after transcatheter re-intervention [23], and 28.2 days after stroke [24]. Informal care costs were assumed to be unchanged after in-hospital treatment of bleeding and prosthetic valve-related events, because care associated with these events is provided in-hospital. After re-intervention, we assumed equal informal care costs as after the initial intervention. After stroke, we assumed that 54% of patients used informal care for 13.5 h/week during the first half year and 8.3 h/week during the second half year and subsequent years [24].

#### Health-related quality of life

Health-related quality of life was expressed in utilities. Utility of patients without complications was measured with the EQ-5D-5L and dependent on patient and intervention characteristics using regression formulas (more details provided in our previous publication [23]). The utility was corrected for events using utility multipliers derived from the literature for a specific time duration after the event based on literature or assumptions (Table 2). Even when patients did not experience events, their utility changed over time due to ageing. During the first 6 years after the intervention, utility was calculated using a regression formula including time-dependent variables [25]. Beyond year six, yearly absolute disutilities of the general population (males:0.00128; females:0.00171) were subtracted from the predicted utility [26].

#### Tissue-engineered heart valves

Exact costs and performance of TEHV are unclear, because TEHV are not yet in clinical use. Therefore, we had to make a number of assumptions on hypothetical TEHV performance and costs, informed by discussions with a research consortium (1Valve) working on the development of in situ TEHV, including biomedical engineers and cardiothoracic surgeons. First, we assumed that TEHV will not be introduced into clinical practice until their safety has been established. For this reason, we did not include any scenarios in which the risks of early mortality or valve-related events were higher than with current heart valve replacements. The procedure to implant TEHV is expected to be comparable to implanting existing heart valve substitutes. Hence, we assumed that early mortality and event risks, which are mainly procedure related and not valve related, are comparable to bioprostheses used for SAVR or TAVI. Further, we assumed that probabilities to die or undergo re-intervention after early and late events were comparable to bioprostheses. Based on expert opinion and aspects on which the ongoing development of TEHV focuses, we investigated three types of improvements that influence the occurrence of late events: (1) Improved *durability* due to lower rates of prosthetic valve dysfunction (SVD and non-structural valve dysfunction; including calcification, structural and residual leak, and thickening of the prosthetic valve) resulting in longer time to re-intervention; (2) Reduced *thrombogenicity*, the tendency of heart valve substitutes in contact with blood to produce a thrombus or clot, resulting in lower rates of prosthetic valve thrombosis and reduced need for anticoagulation treatment; (3) Improved *infection resistance* resulting in lower rates of endocarditis and subsequent hospitalisation and/or re-intervention.

### Analyses

Cost-effectiveness analyses were performed in a Dutch setting from a societal perspective applying a lifetime horizon with costs expressed in 2016 Euros and effects in QALYs. Future health benefits and costs were discounted with 1.5% and 4%, respectively, according to Dutch HTA guidelines [27].

Several scenario analyses were performed to estimate the impact of variations in hypothetical TEHV performance on costs, effects, and cost-effectiveness assuming that the price of TEHV is equal to that of bioprostheses (SAVR:€2500; TAVI:€18,000). First, we performed scenario analyses where durability, thrombogenicity, and infection resistance of TEHV were varied separately with varying rates, compared to bioprostheses. Further, three scenario analyses, in which these three types of improvements were varied simultaneously, were performed (Table 3). In the first combined scenario, the ‘perfect performance’ scenario, we assumed perfect durability, no thrombogenicity, and perfect infection resistance of TEHV in which the occurrence of prosthetic valve-related events was equal to the level in the general population (i.e. zero). In the second combined scenario, the ‘improved performance’ scenario, we assumed improved durability, reduced thrombogenicity, and improved infection resistance of TEHV in which the occurrence of prosthetic valve-related events was reduced by 50% compared to bioprostheses. In the final combined scenario, the ‘partial improved performance’ scenario, we assumed reduced thrombogenicity and improved infection resistance (i.e. events related to thrombogenicity and infection resistance were reduced with 50%), but reduced durability of TEHV (i.e. prosthetic valve dysfunction events increased by 50%) compared to bioprostheses. In all scenarios, occurrence rates of strokes and bleedings were not varied because these events are influenced by anticoagulation treatment which is only prescribed for patients after aortic valve implantation with bioprostheses during the first 3 months after the intervention and is likely to be prescribed for TEHV as well. [3] Hypothetical TEHV were compared to bioprostheses implanted using the same approach: either surgical (SAVR) or transcatheter (TAVI) implantation. In the remainder, SAVR and TAVI refer to the comparator treatment, i.e. heart valve implantations with bioprostheses. The ‘improved performance’ scenario was perceived as the most realistic scenario and was, therefore, used in several additional analyses. Subgroup analyses were performed for patients aged 70–80 and > 80 years for the ‘improved performance’ scenario. For all scenarios, we calculated incremental costs, effects, cost-effectiveness ratio (ICER) and headroom.

**Table 3:** Occurrence of valve-related events with TEHV compared to bioprostheses

Combined scenarios	Long-term valve-related events				
	Prosthetic valve dysfunction (%)	Prosthetic valve thrombosis (%)	Prosthetic valve endocarditis (%)	Stroke	Bleeding
Perfect performance	− **100**	− **100**	− **100**	Equal	Equal
Improved performance	− ***50***	− ***50***	− ***50***	Equal	Equal
Partial improved performance	+ *50*	− ***50***	− ***50***	Equal	Equal

Bold: large improvement in TEHV performance, Bold italic: moderate improvement in TEHV performance, Italic: moderate deterioration in TEHV performance

The headroom is the maximum cost of TEHV to remain cost-effective compared to bioprostheses when applying a cost-per-QALY threshold (SAVR:€20,000; TAVI:€50,000). Different thresholds for SAVR and TAVI were applied, because in The Netherlands, this threshold depends on disease burden with current standard of care; the higher the disease burden, the higher the cost-per-QALY threshold [28]. Disease burden was expressed in proportional shortfall (i.e. fraction of QALYs that people lose relative to their remaining life expectancy when untreated) which can take a value between 0 (minimal burden of disease) and 1 (maximum burden of disease) and was calculated with the iMTA Disease Burden Calculator [29, 30]. The disease burden was 0.19 in SAVR and 0.48 in TAVI patients.

Probabilistic sensitivity analysis (PSA) was performed for the ‘improved performance’ and ‘partially improved performance’ scenarios. PSA was implemented as a double loop: an inner loop, in which 500 patients were sampled with replacement, and an outer loop in which 500 sets of input parameters values of the model were randomly drawn (Supplement 6). For each set of coefficients, mean outcomes over all patients were recorded and the mean and credible interval (i.e. 2.5% and 97.5% percentiles) over all 500 mean values for each outcome were calculated. The incremental costs and effects of hypothetical TEHV compared to existing heart valves were plotted in cost-effectiveness planes and probabilities that the intervention was cost-effective at certain cost-per-QALY thresholds were displayed in cost-effectiveness acceptability curves (CEAC). The results of the PSA are used to calculate the expected value of perfect information (EVPI) for the ‘improved performance’ and ‘partially improved performance’ scenarios. The EVPI is calculated as the difference between the expected value of the decision made assuming perfect information and the decision made using current information [31]. EVPI is presented at the cost-per-QALY threshold for the specific intervention (SAVR: €20,000; TAVI: €50,000).

Budget impact reflects the difference in total population-level costs of SAVR or TAVI with bioprostheses compared to hypothetical TEHV. Budget impact analyses were performed for the ‘improved performance’ scenario for the first 10 years after introduction of TEHV. Differences in population-level costs were calculated by multiplying the differential total costs per patient with the expected number of TEHV candidates, assuming substitution rates of 25, 50, 75 or 100% of bioprostheses by TEHV. The expected annual number of SAVR patients was 1931 patients, based on the average annual number of SAVR recorded in the ACSD between 2007 and 2015. The expected annual number of TAVI patients was 809 or 3745 patients, based on the average annual number of TAVIs recorded in the Dutch health insurance claims database in 2013 and estimations of Durko et al., respectively [4, 5].

### Validation

Extensive internal validation was performed to check the model’s performance using the TECH-VER checklist [32]. External validation was conducted comparing survival and time-to-events derived from our model (applying US survival tables for background mortality [33]) with an external dataset from the US Providence Health System [34] in four subgroups: males and females between 70 and 80 years old and males and females > 80 years old. This Portland dataset contains 2814 patients aged ≥ 70 years who underwent SAVR with bioprostheses with 17,525 follow-up years (mean 6.2 years). We did not have access to an external dataset to validate TAVI outcomes.

## Results

Results of the scenario analyses are presented in Table 4. Of the three TEHV performance components, durability had the highest impact on predicted cost-effectiveness. This is emphasised by the results of the ‘partial improved scenario’ where the consequences of reductions in durability of TEHV for the predicted cost-effectiveness could not be offset by reduction in thrombogenicity and improvement of infection resistance of TEHV. The ‘perfect performance’ scenario provides insight in the maximum predicted lifetime QALY gain and cost savings of TEHV: 0.249 QALYs and €1344 versus SAVR and 0.079 QALYs and €789 versus TAVI. In the ‘improved performance’ scenario, lifetime QALY gains of 0.131 and 0.043, lifetime cost reductions of €639 and €368, translating to headrooms of €3255 and €2498 per TEHV compared to SAVR or TAVI, respectively, were predicted. The median predicted SVD-free life expectancy increased from 9.4 after SAVR to 10.0 years with TEHV and from 4.6 after TAVI to 4.7 years with TEHV (Table S16). Subgroup analyses showed that predicted QALY gain was higher in patients 70–80 years than in patients > 80 years old, while cost reductions were comparable.

**Table 4:** Cost-effectiveness results of scenario analyses

	LY	QALYs	Societal costs	Healthcare costs	∆LYs	∆QALYs	∆Societal costs	∆Healthcare costs	ICER	Headroom (Cost/QALY threshold €20,000;50,000)
*SAVR*										
SAVR with existing valve prostheses	10.196	6.761	150,860	137,447						
Subgroup patients aged 70–80 years	11.047	7.358	159,939	145,852						
Subgroup patients aged > 80 years	6.985	4.488	114,759	103,814						
Improved durability of TEHV										
No prosthetic valve dysfunction events	10.348	6.890	149,440	136,813	0.152	0.129	− 1420	− 634	–	4004
75% less prosthetic valve dysfunction events	10.317	6.861	149,780	136,994	0.121	0.101	− 1080	− 453	–	3094
50% less prosthetic valve dysfunction events	10.280	6.830	150,105	137,132	0.084	0.069	− 756	− 315	–	2134
25% less prosthetic valve dysfunction events	10.245	6.800	150,522	137,329	0.049	0.040	− 338	− 118	–	1130
Reduced thrombogenicity of TEHV										
No valve thrombosis events	10.196	6.761	150,758	137,369	0.001	0.000	− 102	− 79	–	110
75% less valve thrombosis events	10.196	6.761	150,792	137,395	0.000	0.000	− 69	− 53	–	75
50% less valve thrombosis events	10.196	6.761	150,816	137,414	0.000	0.000	− 44	− 33	–	50
25% less valve thrombosis events	10.196	6.761	150,827	137,421	0.000	0.000	− 34	− 26	–	38
Improved infection resistance of TEHV										
No endocarditis events	10.361	6.877	151,118	137,938	0.165	0.116	257	491	2222	2061
75% less endocarditis events	10.323	6.850	151,109	137,865	0.127	0.089	248	418	2792	1530
50% less endocarditis events	10.283	6.821	151,030	137,729	0.087	0.061	170	282	2797	1044
25% less endocarditis events	10.235	6.788	150,889	137,530	0.039	0.028	28	83	1028	526
Perfect durability, no thrombogenicity, and perfect infection resistance (no events)	10.516	7.010	149,517	137,219	0.320	0.249	− 1344	− 228	–	6322
Improved durability, reduced thrombogenicity, and improved infection resistance (50% less events)	10.368	6.892	150,221	137,383	0.172	0.131	− 639	− 65	–	3255
Subgroup patients aged 70–80 years	11.248	7.512	159,397	145,968	0.201	0.154	− 542	115	–	3616
Subgroup patients aged > 80 years	7.065	4.545	114,161	103,436	0.079	0.057	− 598	− 378	–	1740
Decreased durability (50% more events) but reduction in thrombogenicity and improvement in infection resistance (50% less events)	10.220	6.814	152,278	138,452	0.024	0.053	1417	1005	26,841	− 361
*TAVI*										
TAVI with existing valve prostheses	5.679	3.134	100,243	81,202						
Subgroup patients aged 70–80 years	8.000	4.089	120,195	94,010						
Subgroup patients aged > 80 years	4.435	2.630	89,888	74,626						
Improved durability of TEHV										
No prosthetic valve dysfunction events	5.754	3.182	99,665	80,616	0.074	0.048	− 578	− 586	–	1540; 2983
75% less prosthetic valve dysfunction events	5.739	3.172	99,834	80,771	0.060	0.039	− 410	− 430	–	1180; 2335
50% less prosthetic valve dysfunction events	5.721	3.160	99,970	80,902	0.042	0.027	− 273	− 300	–	807; 1608
25% less prosthetic valve dysfunction events	5.702	3.148	100,104	81,045	0.023	0.014	− 139	− 156	–	423; 849
Reduced thrombogenicity of TEHV										
No valve thrombosis events	5.680	3.134	100,121	81,092	0.000	0.000	− 122	− 110	–	126; 132
75% less valve thrombosis events	5.680	3.134	100,156	81,123	0.000	0.000	− 87	− 79	–	91; 97
50% less valve thrombosis events	5.680	3.134	100,180	81,145	0.000	0.000	− 64	− 57	–	66; 69
25% less valve thrombosis events	5.679	3.134	100,208	81,170	0.000	0.000	− 35	− 31	–	35; 35
Improved infection resistance of TEHV										
No endocarditis events	5.742	3.164	100,179	81,074	0.063	0.030	− 64	− 128	–	668; 1574
75% less endocarditis events	5.729	3.157	100,217	81,121	0.050	0.024	− 26	− 81	–	502; 1216
50% less endocarditis events	5.713	3.149	100,218	81,146	0.033	0.016	− 25	− 55	–	341; 815
25% less endocarditis events	5.692	3.140	100,205	81,155	0.013	0.006	− 38	− 47	–	166; 358
Perfect durability, no thrombogenicity, and perfect infection resistance (no events)	5.817	3.213	99,454	80,366	0.137	0.079	− 789	− 836	–	2367; 4734
Improved durability, reduced thrombogenicity, and improved infection resistance (50% less events)	5.755	3.176	99,875	80,786	0.075	0.043	− 368	− 416	–	1220; 2498
Subgroup patients aged 70–80 years	8.156	4.174	119,825	93,526	0.156	0.086	− 370	− 485	–	2082; 4650
Subgroup patients aged > 80 years	4.474	2.652	89,557	74,261	0.038	0.023	− 331	− 365	–	783; 1461
Decreased durability (50% more events) but reduction in thrombogenicity and improvement in infection resistance (50% less events)	5.666	3.139	100,904	81,586	− 0.014	0.005	660	385	122,276; 71,225	− 552; − 390

‘–‘TEHV dominates. *LY* life years, *QALY* quality-adjusted life years, *ICER* incremental cost-effectiveness ratio, *WTP* willingness to pay per QALY gained in Euros, *SAVR* surgical aortic valve replacement, *TAVI* transcatheter aortic valve implantation, *TEHV* tissue-engineered heart valves

**Fig. 2:**
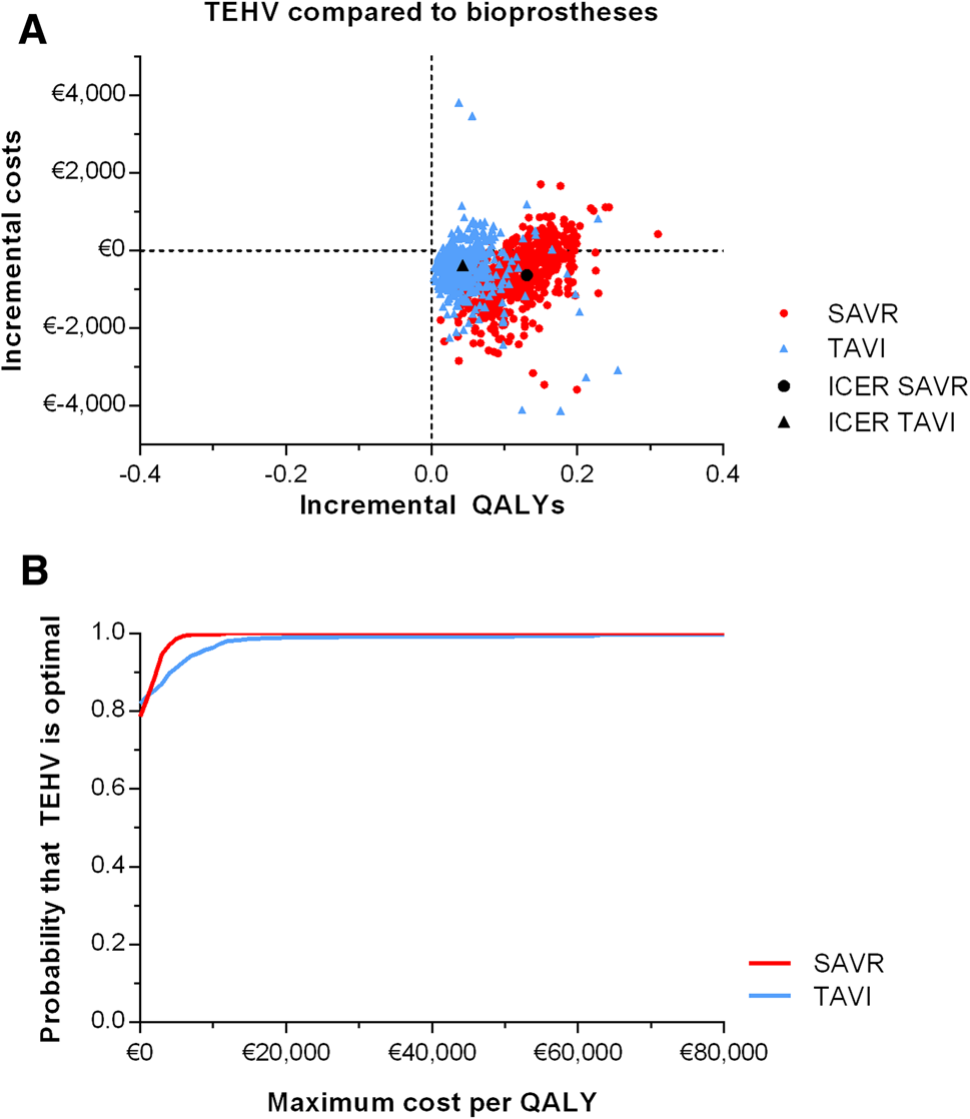
Probabilistic sensitivity analyses outcomes of surgical (SAVR) and transcatheter (TAVI) aortic valve implantation with TEHV (50% improved performance) compared to bioprostheses. **a** Cost-effectiveness plane. **b** Cost-effectiveness acceptability curve (CEAC)

In the PSA of the ‘improved performance’ scenario, predicted incremental costs and effects varied as shown in the cost-effectiveness plane (Fig. 2a), with most data points lying in the south-east quadrant, suggesting QALY gains at lower costs. The CEACs show that when applying a threshold of €20,000 per QALY, there is a probability of cost-effectiveness of 100% for TEHV compared with SAVR and 99% for TEHV compared with TAVI (Fig. 2b). The results of the PSA of the ‘partially improved performance’ scenario are provided in Figure S16. The EVPI in both the ‘improved performance’ and ‘partially improved performance’ scenarios is relatively low. At a willingness to pay threshold of €20,000, the EVPI is €3149 per patient in the ‘improved performance scenario’ and €60 for in the ‘partially improved performance scenario’ for TEHV compared to SAVR. At a willingness to pay threshold of €50,000, the EVPI is €2873 per patient in the ‘improved performance scenario’ and €119 in the ‘partially improved performance scenario’ for TEHV compared to TAVI. This can be explained by the fact that there is low uncertainty about the cost-effectiveness of TEHV in the ‘improved performance’ scenario (i.e. high probability of being cost-effective) and in the ‘partially improved performance’ scenario (i.e. low probability of being cost-effective). As a result, additional information on the input parameters is unlikely to change the reimbursement decision.

**Fig. 3:**
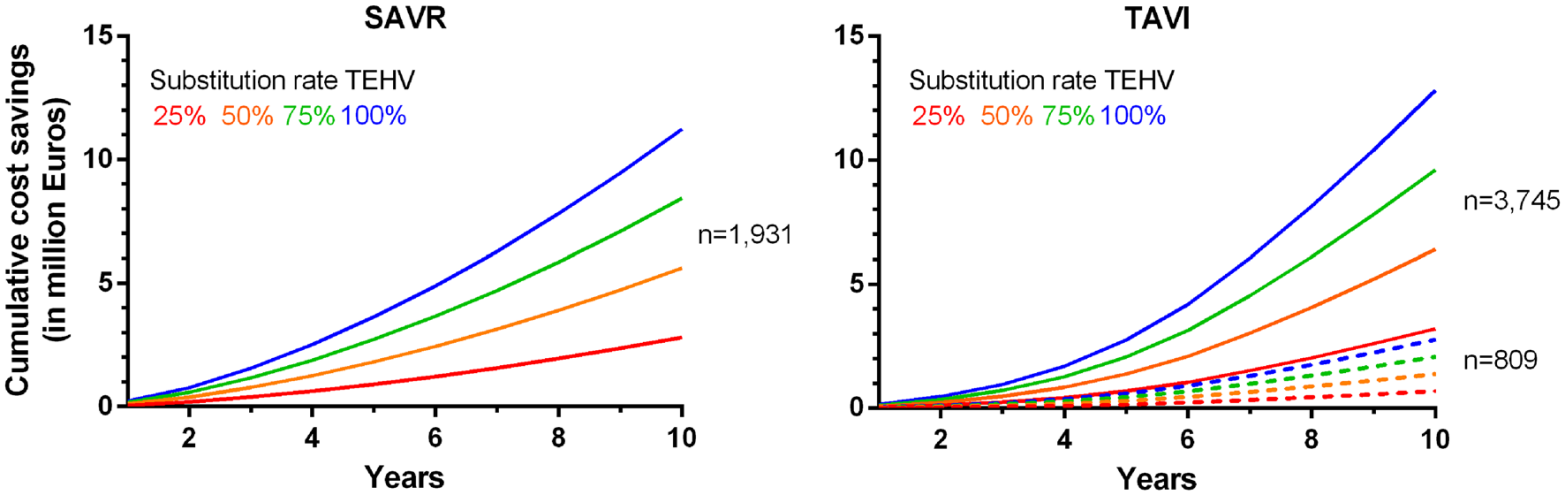
Cumulative cost savings in the first 10 years after introduction of surgical (SAVR; left) and transcatheter (TAVI; right) aortic valve implantation with TEHV (‘improved performance’ scenario) compared to bioprostheses

Figure 3 (Table S17) shows that implementing SAVR and TAVI with TEHV (in the ‘improved performance’ scenario) instead of bioprostheses resulted in predicted cost savings of the Dutch healthcare budget in the next 10 years varying between €2.8–€11.2 (SAVR) and €3.2–€12.8(TAVI) million, for TEHV substitution rates of 25% or 100%.

The predicted headroom of TEHV compared to SAVR and TAVI, respectively, varied from €38 and 35 if TEHV would only result in a small reduction in thrombogenicity to €6322 and 4734 if there would be no prosthetic valve-related events at all using TEHV (Table 4).

Extensive internal validation was performed to check the model’s performance using the TECH-VER checklist [32]. Further, Kaplan–Meier curves of survival and time to SVD that were used as input were comparable to curves derived from the model (Supplement 8) [8]. External validation of the model’s survival output with the Portland dataset showed that results were comparable, but the model predicted a slightly higher survival, especially in females between 70 and 80 years old (Supplement 9). There were discrepancies between cumulative incidence functions of events; the number of events in the model was higher than observed in Portland (Supplement 9).

## Discussion

This early HTA study showed that hypothetical TEHV are likely to be cost-effective when used in elderly patients with aortic valve disease, under the current assumptions about improvements, compared to bioprostheses [2]. Improvements in durability of TEHV had the greatest impact on cost-effectiveness. Improved durability not only increased lifetime QALYs, but also reduced costs. In addition, it is worthwhile to pursue improved infection resistance, considering the lifetime QALY gains that can be achieved with acceptable costs when prosthetic valve endocarditis is prevented. Reductions in thrombogenicity are unlikely to significantly impact cost-effectiveness of TEHV in elderly patients. In these patients, reduced thrombogenicity only reduces valve thrombosis occurrence, since lifelong anticoagulation treatment is not required with bioprostheses or patients already use anticoagulation treatment for other indications (resulting in less strokes and more bleedings than in the general population) [8]. However, reduced thrombogenicity may have more impact on cost-effectiveness of TEHV in younger patients who are eligible for mechanical valves and often have no other indication for anticoagulation treatment as reduced thrombogenicity would not only reduce valve thrombosis and strokes, but also the need of lifelong anticoagulation associated with increased bleedings. Finally, subgroup analyses showed that benefits of TEHV are lower in older patients (> 80 years), because their lifetime risks on events and subsequent re-intervention are lower due to their shorter life expectancy.

Due to the high prevalence of aortic valve disease in elderly patients, using TEHV instead of bioprostheses may lead to costs savings of more than €10 million in the next decade in The Netherlands. However, cost savings and QALY gains per individual patient were relatively low. Higher individual cost savings and QALY gains may be achieved when TEHV are used in younger patients as more benefits can be gained during their remaining life expectancy.

The magnitude of national cost savings depends on perspective, substitution rate of TEHV, and patient population size. Although TEHV may eventually become the gold standard heart valve substitute, it is more likely that substitution will increase gradually, as observed in the adoption of TAVI in Western Europe where four years after introduction only 17.9% of potential candidates underwent TAVI [35]. Further, the actual cost savings might be higher than reported in this study, because our estimates were not adjusted for the expected increase in aortic valve implantations due to ageing of the general population [2, 6].

Considering the QALY gains TEHV might achieve, TEHV may be sold at a higher price and still remain cost-effective compared to bioprostheses. Depending on TEHV performance, the headroom per heart valve substitute (assumption: TEHV price is equal to bioprostheses; i.e. SAVR: €2500; TAVI: €18,000) varied between €38 and €6323 compared with SAVR and €35-€4734 compared with TAVI. When we also applied a cost-per-QALY threshold of €20.000 for TAVI, the headroom varied between €35 and €2367 per heart valve substitute. Instead of assuming the price of TEHV is equal to bioprostheses, it may be expected that manufacturing costs will be comparable to other inorganic heart valve substitutes (i.e. mechanical valves, €1500). Considering these relatively low expected manufacturing costs, the results of our headroom analyses are even more promising for the commercial viability of TEHV.

External validation of model outcomes with actual survival and event data from the Providence Health System showed that the observed survival was slightly lower than the model’s predicted survival. Possible explanations can be the slightly lower mean age and considerably higher concomitant CABG proportion in patients in the Portland dataset compared to the model (Table S16). Survival difference was larger in females between 70 and 80 years old than in other subgroups. This can be explained by comparable survival of 70- to 80-year-old females and males in the Portland dataset, while a higher survival of females than males was applied in the model as observed in the general population [18]. Further, cumulative incidence of valve-related events was lower in the Portland dataset than in the model. Explanations for this discrepancy may be overestimation of occurrence of events in the meta-analyses as zero events may not be reported in the published literature, underreporting of events in the Portland dataset, too short follow-up of patients because mean event-free life expectancy in the model was higher than mean follow-up of patients in the Portland dataset, or differences in outcomes between The Netherlands and the US.

Inherent to any early HTA, we had to make assumptions regarding costs and clinical performance of TEHV. Therefore, this study presents a theoretical exercise and the results are a prediction of the potential cost-effectiveness of hypothetical TEHV. It is currently uncertain if and when TEHV will be introduced in clinical practice and whether the performance will indeed be improved compared to bioprostheses. However, it is becoming more likely that TEHV will have this improved performance, because preclinical and first-in-man clinical trials of TEHV and vascular grafts showed promising results and recently a small-scale first-in-man clinical trial of tissue-engineered pulmonary valved conduits for children was initiated [10, 12]. However, there are still several unresolved challenges regarding heart valve tissue engineering, including finding the optimal material for the scaffold [36], the induction of regeneration of functional tissue [9], and finding the optimal balance between scaffold degradation and the formation of new tissue [9].

There are only a small number of publications on early HTA of tissue-engineered therapies. In addition to this analysis, we also performed an early HTA of TEHV in children requiring pulmonary valve replacement (PVR) [37]. The cost-effectiveness outcomes are difficult to compare due to methodological differences between the analyses, but in general, the cost savings associated with TEHV were considerably smaller in elderly requiring SAVR/TAVI than in children requiring PVR due to the relatively low probability of re-intervention in elderly patients compared to children. Furthermore, Tan et al. performed a cost-minimization analysis for comparing tissue-engineered constructs to donor tissue procured from eye banks for endothelial keratoplasty [38]. They concluded that the tissue engineering strategy was cheaper in both investment cost (i.e. costs of the capital outlay for the necessary equipment) and recurring cost (manufacturing cost per construct) [38]. In our study, the costs of manufacturing TEHVs were not calculated in detail but assumed to be equal to currently used bioprostheses. The analysis of Tan et al. suggest, however, that the costs of TEHV may be lower, which increases the likelihood of TEHV being cost-effectiveness even more.

This study has several limitations. Firstly, we strived to use the memory of our patient-level simulation model to make survival, valve-related events, utilities and costs dependent on patient and intervention characteristics and patient history (i.e. previous events). We did indeed do so for early mortality, early clinical outcomes, costs and utilities. However, relationships between occurrence rates of valve-related events after aortic valve implantation on the one hand and patient and intervention characteristics and history of previous valve-related events on the other hand remain poorly defined and could, thus, not be incorporated into our model. Secondly, the model requires assumptions about evolution of event occurrence rates and hazard ratio for excess mortality beyond the observed follow-up period, which introduced uncertainty in the extrapolation of these events. Thirdly, most healthcare cost estimates were based on health insurances claims data which means that, conflicting with the applied societal perspective, costs represent expenditures reimbursed by health insurers based on agreements between healthcare providers and insurers, not actual costs. Fourthly, this study was performed from a Dutch perspective and may, therefore, not be generalizable to other countries. Finally, additional informal care use or productivity loss after events, except for stroke and re-intervention, were not incorporated due to limited data availability. However, additional informal care use and productivity loss of patients after hospitalisation for these events are probably relatively low and, therefore, will not have a large impact on total costs.

The results of this study can be useful for different stakeholders. First, we informed engineers about minimum performance requirements and maximum additional costs of TEHV to be cost-effective compared to bioprostheses early in the development process [39]. We showed that engineers should primarily focus on durability. Further, a higher price for TEHV than bioprostheses is possible. Second, it provides patients and clinicians the first estimates of potential improvements in clinical outcomes of TEHV, which may result in faster adoption of TEHV in clinical practice [40]. We showed that, although benefits of TEHV may be relatively low in elderly patients due to their limited remaining life expectancy, TEHV may result in improvements in (quality-adjusted) life expectancy and reduced costs. Finally, this study informs Dutch healthcare payers about the possible entrance of TEHV to the market and its associated national cost savings, which may result in more timely decisions about reimbursement [40].

In conclusion, when biomedical engineers succeed in realising improved durability and/or infection resistance of TEHV in the aortic position in elderly patients, TEHV have the potential to be cost-effective compared to both surgical and transcatheter bioprostheses and commercially viable in this patient group. Due to the relatively short life expectancy of elderly patients undergoing aortic valve implantation, individual cost savings and QALY gains are relatively meagre, but due to the large size of the patient population, national cost savings can become substantial.

## Electronic supplementary material

Below is the link to the electronic supplementary material.

Supplementary material 1 (PDF 1012 kb)
