# Supplementary material for: Early cost-utility analysis of tissue-engineered heart valves compared to bioprostheses in the aortic position in elderly patients
Source: Eur J Health Econ. 2020 Jan 25;21(4):557–72. doi: 10.1007/s10198-020-01159-y (PMC7214484; doi:10.1007/s10198-020-01159-y)
Supplement: Supplementary file 1 — Supplementary material 1 (PDF 1012 kb) [file 10198_2020_1159_MOESM1_ESM.pdf]

Supplemental Material – Early Health Technology Assessment of Tissue-Engineered Heart Valves in the Aortic Position in Elderly Patients

Met opmerkingen [SH1]: Volgorde table nummers en CHEERS checklist toevoegen

Supplement 1. Baseline patient population

Surgical aortic valve replacement (SAVR)

The database used for sampling with replacement is the Adult Cardiac Surgery Database (ACSD) from The Netherlands Association for Cardio-Thoracic Surgery (NVT). This database includes patient- and intervention characteristics, early mortality, and several peri- and postoperative complications of 40,213 patients who underwent heart valve replacement between 2007 and 2015 in the Netherlands. The database included almost all patient- and intervention characteristics needed to calculate patient-specific early mortality and event risks, utilities, and costs, except for comorbidities and several concomitant procedures.

For the calculation of costs, comorbidities are divided in four groups: (1) COPD, DM, kidney injury and/or HF, (2) none of the comorbidities in group 1 and hypertension, (3) other comorbidities than group 1 and 2, and (4) no comorbidities. The ACSD only includes data on COPD and diabetes mellitus. The remaining patients were randomly assigned to one of the comorbidity subgroups based on age group specific proportions derived from a healthcare insurance claims database.[1] This database contains the healthcare expenditures of all the insured who underwent heart valve replacement between 2010 and 2013 in the Netherlands. The randomly assigned comorbidity variable will only be used for costs calculations, not for determining clinical outcomes or utilities.

The following concomitant procedures included in the regression formula for intervention costs derived from the health insurance claims database were not available in the ACSD: correction of Tetralogy of Fallot, hypertrophic obstructive cardiomyopathy (HOCM), and left ventricle repair.

In total 35,258 patients did not have missing values in the required variables (Table S2). The patient and intervention characteristics of the 15,405 patients above 70 years old who underwent aortic valve replacement we have selected for this analysis are presented in Table S1.

Transcatheter aortic valve implantation (TAVI)

Unfortunately we did not have access to a clinical dataset such as the ACSD for patients after TAVI. Instead we have simulated a patient population using patient characteristic frequencies from the health insurance claims database.[1] The Vektis database is a health insurance claims database which contains the healthcare expenditures of all the insured in the Netherlands. The patient and intervention characteristics of the 809 patients ≥70 years old who underwent TAVI between 2010-2013 are presented in Table S1.

Table S1. Baseline characteristics of patient populations

BASELINE CHARACTERISTICS	SAVR (n=15,405)	TAVI (n=809)
**Patient related**		
**Age, mean±SD (range)**	77.0±4.1 (71-94)	81.9±4.9 (70-94)
**Gender (%)**	55.03	47.84
**Previous cardiac surgery (%)**	7.81	-
**Previous valve replacement[1]**	-	-
**Preoperative serum creatinine level > 200 µmol/l (%)**	1.79	-
**LV function (%)**		
LVEF >50%	77.74	-
LVEF 30-50%	18.42	-
LVEF <30%	3.84	-
COPD (%)	15.29	-
**Peripheral vascular disease (PVD) (%)**	13.29	-
**Neurological dysfunction(%)**	2.80	-
**Previous CVA[1]**	-	-
**Preoperative endocarditis (%)**	2.06	-
**Instable angina pectoris (%)**	1.06	-
**Pulmonary hypertension (%)**	2.99	-
**Co-morbidity categories in cost-analyses (%)**		
- COPD, DM, kidney disease and/or HF	53.04	64.03
- Hypertension	31.86	24.60
- Other co-morbidities	5.90	4.45
- No co-morbidities	9.20	6.92
**Socioeconomic status (SES, in quartiles) (%)**		
0-20	23.62	24.97
21-40	25.64	19.90
41-70	26.35	28.18
71-100	24.39	26.95
**Procedure related**		
**Type of valve prosthesis (biological or mechanical)[2]**	-	-
**Emergency procedure (%)**	2.03	-
**Concomitant procedures (%)**		
- CABG	44.48	-
- Other valve repair	7.69	
- Thoracic aorta surgery	5.84	-
- Bentall procedure	1.51	
- Aorta ascendens procedure	0.90	-
- MAZE	0.40	-
- Aortic arch procedure	0.40	-
- Aortic root procedure	0.39	-
- Aorta descendens procedure	0.08	-
- Other valve replacement	0.04	-

LV: left ventricle. LVEF: left ventricular ejection fraction. COPD: chronic obstructive pulmonary disease. CVA: cerebrovascular accident. DM: diabetes mellitus. HF: heart failure. CABG: coronary artery bypass grafting. MAZE: heart surgery for atrial fibrillation. QALY: quality adjusted life year. [1]We assumed all patients in the starting population did not have previous valve replacement or CVA because it was not available or there were many missing values in the database. [2]Not available in the database, but considering the high age of these patients we assume they all received a bioprosthesis.

Supplement 2 – Changes in conceptual model of Huygens et al. 2016.

The background of this study and the development of the conceptual model is described before.[2] This supplement describes the changes that were made to the conceptual model described by Huygens et al.[2] To be in line with the definition in the ACSD and published literature, the definition of cerebrovascular accident was changed to stroke for both SAVR and TAVI. In addition, atrial fibrillation and acute kidney injury were changed into arrhythmias and renal failure for SAVR patients to be in line with the definition used in the ACSD. Finally, conversion to another approach (transcatheter to surgical valve implantation and vice versa) was excluded from the final decision analytic model. Emergent conversion from TAVI to SAVR occurs rarely (1.2%-2.1%) and according to expert opinion conversion from SAVR to TAVI occurs even less.[3, 4] In addition, since the causes of conversion of approach are not related to the prosthetic heart valve itself, the conversion rate is likely to be comparable for TEHV and existing heart valve substitutes.[4]

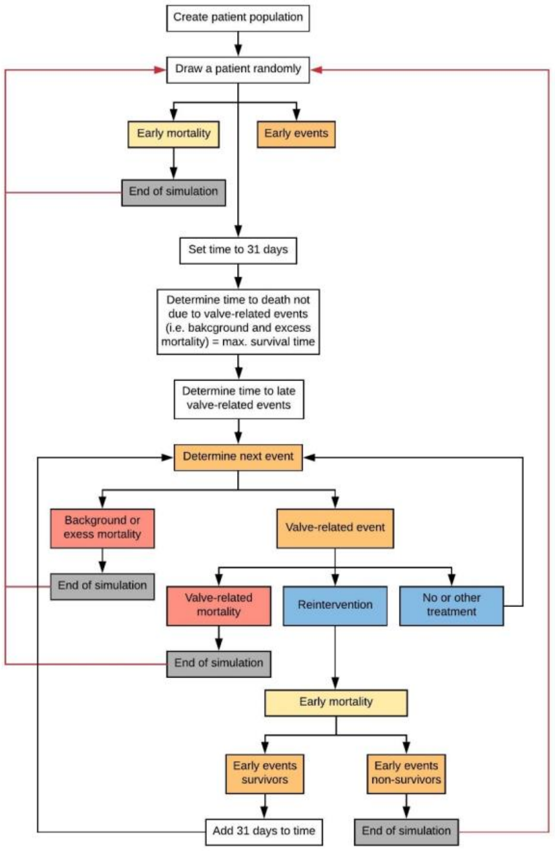

Figure S1. Flowchart patient level simulation model

Table S2. Definitions of parameters

BASELINE CHARACTERISTICS	
**Patient related**	
Age*	Continuous
Sex	Male = 1; female = 0
Previous cardiac surgery*	Previous cardiac surgery in which the pericardium was opened.
Previous valve replacement*	Previous surgery where the heart valve was replaced.
Preoperative serum creatinine level > 200 µmol/l	The last recorded preoperative serum creatinine level of the blood was higher than 200 µmol/l.
Left ventricular (LV) function	The percentage of the end-systolic volume of the blood in the left ventricle with respect to the final diastolic volume. Higher left ventricle ejection fraction (LVEF), reflects better LV function.
Chronic obstructive pulmonary disease (COPD)	Chronic obstructive pulmonary disease
Peripheral vascular disease (PVD)	When one or more of the following criteria are fulfilled:<br>- Claudication;<br>- Carotid occlusion or >50% stenosis;<br>- Amputation due to arterial disease;<br>- Previous or planned surgery on abdominal aorta, arteries of the limbs or carotids.
Neurological dysfunction	Disease that impairs daily functioning severely.
Previous cerebrovascular accident (CVA)*	History of CVA with or without residual injury.
Preoperative endocarditis*	At the moment of the heart valve replacement the patient is being treated with antibiotics for endocarditis.
Instable angina pectoris	Angina pectoris that requires intravenous nitrate therapy until entering the operation theatre.
Pulmonary hypertension	Condition of increased blood pressure within the arteries of the lungs.
Co-morbidity categories in cost-analyses	Co-morbidities were based on Pharmacy Cost Groups, which is an outpatient morbidity measure based on prior use of prescribed drugs as marker for chronic conditions.
- COPD, diabetes mellitus (DM), kidney disease and/or heart failure (HF)	Patients with COPD, DM, kidney disease and/or HF.
- Hypertension	Patients without COPD, DM, kidney disease and/or HF, but with hypertension.
- Other co-morbidities	Patients with other co-morbidities than COPD, DM, kidney disease, HF or hypertension.
- No co-morbidities	Patients without co-morbidities.
Socioeconomic status	Based on status scores reflecting the SES of a district (defined by postal code) based on characteristics of its residents: education, income, and position on the labor market. The status scores were divided in four groups based on percentiles, with lower percentiles representing lower SES.
**Procedure related**	
Emergency procedure	Unplanned intervention that cannot wait until the beginning of the next working day due to medical reasons.
Concomitant procedures	Procedures that are performed at the same time of the valve replacement.
- Coronary artery bypass grafting (CABG)	Coronary artery bypass grafting.
- Other valve replacement	Replacement of more than one valve.
- Other valve repair	Repair of another valve than the valve being replaced.
- Aortic root procedure	Intervention on the aortic root (part of the aorta from the aortic valve until the sinotubular junction) only.
- Aorta ascendens procedure	Intervention on the aorta ascendens (part of the aorta from the aortic valve until the arteria anonyma) only.

- Bentall procedure	Intervention involving composite graft replacement of the aortic valve, aortic root and ascending aorta, with re-implantation of the coronary arteries into the graft.
- Aortic arch procedure	Intervention on the aortic arch (part of the aorta beyond the arteria anonyma until the arteria subclavia sinistra).
- Aorta descendens procedure	Intervention on the aorta (part of the aorta beyond the subclavian sinister artery until beyond the diaphragm.
- Thoracic aorta surgery	Surgical intervention on the aorta ascendens, arch or descendens.
- Maze procedure	Surgical treatment for atrial fibrillation.
**EVENTS**	
Stroke	Stroke with or without residual injury.
Myocardial infarction (MI)†	Perioperative myocardial infarction (MI). MIs were registered according to the definition used in the STS Adult Cardiac Surgery Database.[5]
Vascular complication†	All arterial vascular complications, such as dissection of the aorta, acute ischemia of the arm or leg due to vascular problems, IABP complications, etc.
Bleeding	Any episode of major internal or external bleeding that causes death, hospitalization, or permanent injury (e.g., vision loss) or necessitates transfusion.[6]
Atrial fibrillation (AF)/arrhythmias†	In the Adult Cardiac Surgery database, atrial fibrillations were not separately recorded. Therefore we used the registrations of arrhythmias including all forms of arrhythmia requiring treatment (such as resuscitation because of cardiac arrest or new onset atrium fibrillation or flutter that necessitates intervention (defibrillation or medication)). Spontaneous transient periods of atrial fibrillation without any consequence for the patient were not registered. Costs and utilities were based on atrial fibrillations, instead of all arrhythmias.
Pacemaker implantation (PI)†	Implantation of a medical device that uses electrical impulses, delivered by electrodes contracting the heart muscles, to regulate the beating of the heart.
Acute kidney injury (AKI)†	Renal failure was registered in the Adult Cardiac Surgery Database if one or more of the following criteria were fulfilled during the postoperative period: renal replacement treatment (dialysis, CVVH) not existing before procedure and/or highest postoperative serum creatinine level > 177 μmol/L and doubled preoperative level. This narrow definition does not include acute kidney injury stage 1 as defined by the AKIN classification in VARC-2.[7]
Structural valve deterioration (SVD)	Dysfunction or deterioration involving the operated valve (exclusive of infection or thrombosis), referring to changes intrinsic to the valve, such as wear, fracture, poppet escape, calcification, leaflet tear, stent creep, and suture line disruption of components of a prosthetic valve.[6]
Non-structural valve dysfunction (NSD)	Any abnormality not intrinsic to the valve itself that results in stenosis or regurgitation of the operated valve or hemolysis.[6]
Prosthetic valve thrombosis	Any thrombus not caused by infection attached to or near an operated valve that occludes part of the blood flow path, interferes with valve function, or is sufficiently large to warrant treatment.[6]
Prosthetic valve endocarditis	Any infection involving a prosthetic valve.[6]
Reintervention	Any surgical or transcatheter procedure that repairs, otherwise alters or adjusts, or replaces a previously implanted prosthesis.[6]

*Not stable over time. †Events are only included during the first thirty days after the intervention.

Table S3. Specification of type of equation per outcome

Equation number	Outcome	Type of equation
**Early mortality after SAVR**		
1	Probability of early mortality	Generalized linear model with binominal family (glm function in R)<br><br>Probability early mortality = age + male gender + previous cardiac surgery + preoperative serum creatinine level > 200 µmol/l + LVEF 30-50% + LVEF <30% + COPD + PVD + neurological dysfunction + emergency procedure + concomitant CABG + concomitant aorta root procedure + concomitant Bentall procedure + concomitant aorta ascendens procedure.
**Early events after SAVR** (separate models with same predictor variables for patients that survive or do not survive the first 30 days after the intervention)		
2	Probability of early CVA	Generalized linear model with binominal family (glm function in R)<br><br>Probability early CVA = age + male gender + previous CVA + previous cardiac surgery + preoperative serum creatinine level > 200 µmol/l + LVEF 30-50% + LVEF <30% + COPD + PVD + neurological dysfunction + instable angina pectoris + pulmonary hypertension + preoperative endocarditis + emergency procedure + concomitant other valve replacement + concomitant CABG + concomitant aorta ascendens procedure.
3	Probability of early renal failure	Generalized linear model with binominal family (glm function in R)<br><br>Probability early renal failure = age + male gender + previous CVA + previous cardiac surgery + preoperative serum creatinine level > 200 µmol/l + LVEF 30-50% + LVEF <30% + COPD + PVD + emergency procedure + concomitant CABG.
4	Probability of early arrhythmias	Generalized linear model with binominal family (glm function in R)<br><br>Probability early arrhythmias = age + male gender + previous cardiac surgery + preoperative serum creatinine level > 200 µmol/l + LVEF 30-50% + LVEF <30% + COPD + PVD + emergency procedure + concomitant other valve replacement + concomitant CABG + concomitant aorta ascendens procedure + concomitant aorta descendens procedure + concomitant aorta arch procedure.
5	Probability of early MI	Generalized linear model with binominal family (glm function in R)<br><br>Probability early MI = age + male gender + previous cardiac surgery + preoperative serum creatinine level > 200 µmol/l + LVEF 30-50% + LVEF <30% + PVD + emergency procedure + concomitant other valve replacement + concomitant CABG.

**Healthcare costs**		
6	Intervention costs	Generalized linear model with gamma distribution and identity link (PROC GENMOD in SAS)<br><br>Intervention costs = valve position + concomitant procedures + age group + male gender + co-morbidity category + SES class + death within 6 months after the intervention.
7	Event costs (AKI, AF, stroke, MI, PI, reintervention)	Generalized linear model with gamma distribution and identity link (PROC GENMOD in SAS)<br><br>Event costs = age group + male gender + co-morbidity category + SES class + death within 6 months after the complication.
8	Other healthcare costs	Multilevel generalized linear model for with normal distribution and identity link (PROC GLIMMIX in SAS)<br><br>Other healthcare costs adults = time since intervention + death + age group at intervention + male gender + SES class + AF + AKI + stroke + TIA + endocarditis + MI + PI + reintervention.<br><br>Other healthcare costs children = time since intervention + male gender + SES class.
**Societal costs**		
9	Productivity costs of unpaid work	Generalized linear models with binominal family (glm function in R)<br><br>Probability of unpaid work after SAVR = age + male + years since intervention + biological valve (compared to mechanical valve) + concomitant CABG + multiple valve replacement.<br><br>Probability of unpaid work after TAVI = age + male + years since intervention<br><br>Probability of less unpaid work after SAVR = age + male + years since intervention + biological valve (compared to mechanical valve) + concomitant CABG + multiple valve replacement.<br><br>Probability of less unpaid work after TAVI = age + male + years since intervention + biological valve (compared to mechanical valve) + concomitant CABG + multiple valve replacement.<br><br>Productivity costs unpaid work last four weeks after SAVR = age + male + years since intervention + biological valve (compared to mechanical valve) + concomitant CABG + multiple valve replacement.<br><br>Generalized linear model with gamma family and log link (glm function in R)<br><br>Estimated productivity costs last four weeks = probability of unpaid work * probability of less unpaid work * estimated productivity costs of less unpaid work
10	Informal care costs	Probability of using informal care after SAVR = age + male + years since intervention + biological valve (compared to mechanical valve) + concomitant CABG + multiple valve replacement.<br><br>Probability of using informal care after TAVI = age + male + years since intervention

		Generalized linear model with gamma family and inverse link (glm function in R)<br><br>Informal care costs per week after SAVR = age + male + years since intervention + biological valve (compared to mechanical valve) + concomitant CABG + multiple valve replacement.<br><br>Informal care costs per week after TAVI = age + male + years since intervention<br><br>Estimated informal care cost per week = probability of using informal care * informal care costs per week
**Utilities**		
12	Probability of having utility 1	Generalized linear model with binominal family (glm function in R)<br><br>Probability utility of 1 after SAVR = age + male sex + years since SAVR + biological valve prosthesis + concomitant CABG + concomitant other valve replacement + previous valve replacement<br><br>Probability utility of 1 after TAVI = age + male sex + years since TAVI + transfemoral approach
13	Utility below 1	Generalized linear model with gamma family and log link (glm function in R)<br><br>Utility below 1 after SAVR = age + male sex + years since SAVR + biological valve prosthesis + concomitant CABG + concomitant other valve replacement + previous valve replacement<br><br>Utility below 1 after TAVI = age + male sex + years since TAVI + transfemoral approach

Supplement 3 – Excess mortality

Excess mortality is expressed as a hazard ratio of the additional excess mortality not directly resulting from valve-related events relative to background mortality. The estimation of this hazard ratio in elderly patients after SAVR was reported previously.[8] For the estimation of this hazard ratio in TAVI patients, the model containing only background mortality and mortality due to valve-related events (excluding early mortality) was run for 10,000 iterations at the mean age and proportion of males of the UK TAVI registry.[9] Subsequently, the hazard ratio was estimated by fitting the survival output of this simulation model to the survival observed in the UK TAVI registry (excluding early mortality) using varying values for the hazard ratio of excess mortality. The best fit was determined by using the least squares method (Table S4).

Table S4. Least squares regression of modeled survival vs. observed survival for estimation of excess mortality not directly related to valve-related events.

Hazard ratio[1]	Sum of squared residuals[2]
0.9	6200
1.0	4141
1.4	268
**1.5**	**113**
1.6	197
1.7	493
2.0	2341

Bold print indicates the selected model. [1]Hazard ratio of background mortality + excess mortality relative to background mortality. [2]Sum of squared residuals between microsimulation-based survival and survival observed in our meta-analysis of Kaplan-Meier freedom from all-cause mortality.

Supplement 4 – Clinical input parameters

Surgical aortic valve replacement (SAVR)

Early mortality

The data on early mortality and events after SAVR was derived from the Adult Cardiac Surgery Database (ACSD) from The Netherlands Association for Cardio-Thoracic Surgery (NVT). This database includes patient and intervention characteristics, early mortality (i.e. death within 30 days after the intervention), and several peri- and postoperative complications (CVA, renal failure, vascular complications, rhythm problems and myocardial infarction (only perioperative)). For the logistic regression analysis of early mortality we extracted the records of aortic valve replacements (AVR) from 1 January 2007 until 31 December 2015. In total there were 35,732 (isolated or combined) AVR procedures in the Netherlands.

Previously, we identified the potentially relevant predictors of in-hospital events and mortality in patients with heart valve disease[2]: age, gender, symptomatic status (New York Heart Association class), left ventricular ejection fraction (3 categories), pulmonary artery systolic pressure, creatinine (< or > 200), chronic pulmonary disease, extracardiac arteriopathy/peripheral vascular disease, neurological impairment affecting daily activity, concomitant coronary artery disease, concomitant coronary artery bypass surgery, type of valve surgery, concomitant surgery of the ascending aorta, redo cardiac surgery, emergency surgery, frailty, major organ system dysfunction, and procedure-specific impediments. Some of these predictors were not available in the ACSD: concomitant coronary artery disease, frailty, major organ system dysfunction and procedure specific impediments. Further, NYHA class and pulmonary artery systolic pressure were available in the ACSD but the proportion of missing values was very high (>55%) and therefore these predictors were not included in the regression analysis to predict early mortality and early events.

There were missing values for the following variables: gender (n=1, 0.0%), previous cardiac surgery (n=3,057, 8.6%), preoperative serum creatinine level > 200 µmol/ (n=347, 1.0%), LV function (n=78, 0.2%), COPD (n=52, 0.1%), peripheral vascular disease (n=52, 0.1%), neurological dysfunction (n=47, 0.1%), and emergency surgery (n=48, 0.1%). There were 32,345 (90.5%) complete case and 3,387 uncomplete cases. Table S5 compares the patient- and procedure related risk factors, peri- and postoperative complications, and early mortality of complete cases with cases with at least one missing value. The cases with one or more missing value are younger, more often have a serum creatinine level above 200 µmol/l, higher LV ejection fraction, less peripheral vascular disease, and less concomitant procedures. Early mortality risk is slightly lower in the uncomplete cases.

Variables with missing values were completed by multiple imputation (MI). We assume these values were Missing at Random (MAR). MI was performed with the Amelia package. We constructed 50 imputed datasets.[10] We included all available variables in the imputation model, except for the variables that had too many missing values (>80%) within the subgroup of incomplete cases, unless these variables were to be included in the logistic regression analysis of the data after imputation (previous cardiac surgery) (Table S6).

Subsequently, logistic regression analyses were performed with the glm function in R for every imputed dataset. Table S7 shows the pooled estimates of these analyses compared to the logistic regression analysis of only the complete cases. In the final model we used the equation based on the imputed cases.

Early events

Table S8-11 show the logistic regression formulas for the risk on early events specific for patients who survive and those who do not survive the first 30 days after SAVR. Since there were many missing values in the occurrence of early events (>50%), the logistic regression analyses are based on the complete cases. The area under the curve of these formulas are low, but we decided to implement these formulas in the model anyway to make use of the available data.

The occurrence of the other early events (vascular complication, bleeding, pacemaker implantation, prosthetic valve dysfunction, valve thrombosis and endocarditis) was not available in the ACSD or only a small number of events occurred. The risk on reexploration for bleeding (4.2%) and pacemaker implantation (8.1%) was derived from our meta-analysis outcomes after AVR with bioprostheses in elderly patients.[8] We assume this risk is equal for all patients. The other early event risks were assumed to be zero for all patients.

Stroke (Table S8)

The following possible predictors for stroke after AVR were identified from previous studies investigating predictors of stroke after cardiac surgery: age, gender, previous CVA, previous cardiac surgery, isolated vs. Concomitant other valve replacement, concomitant CABG, concomitant aorta ascendens surgery, serum creatinine level > 200 µmol/l, LV function, COPD, diabetes, extra cardiac arterial vascular pathology, neurological dysfunction, preoperative endocarditis, emergency procedure, instable angina pectoris, and pulmonary hypertension.[11-13] Unfortunately, for diabetes the proportion of missing values in the Adult Cardiac Surgery Databases was very high (>55%) and therefore this variable was excluded from the analyses. The tables below show the results of the logistic regression analyses of stroke for patients that do and do not survive the first 30 days.

Renal failure (Table S9)

Renal failure was registered in the Adult Cardiac Surgery Database if one or more of the following criteria were fulfilled during the postoperative period: renal replacement treatment (dialysis, CVVH) not existing before procedure and/or highest postoperative serum creatinine level > 177 µmol/L and doubled preoperative level. This narrow definition does not include acute kidney injury stage 1 as defined by the AKIN classification in VARC-2.[9]

The following variables were identified from previous studies investigating predictors of renal failure after cardiac surgery: age, gender, preoperative renal insufficiency, prior cardiac surgery, NYHA class, congestive heart failure, LV function, peripheral vascular disease, COPD, diabetes, preoperative intra-aortic balloon pump, emergency procedure, concomitant CABG, and increased cardiopulmonary bypass time.[10] Perioperative renal insufficiency was operationalized with the dummy variable serum creatinine level above or below 200 µmol/l. NYHA class and diabetes were available in the Adult Cardiac Surgery Database but the proportion of missing values was very high (>55%) and therefore these variables were excluded from the analyses. Congestive heart failure, preoperative intra-aortic balloon pump, and increased cardiopulmonary bypass time were not available in the Adult Cardiac Surgery Database. The tables below show the results of the logistic regression analyses of renal failure for patients that do and do not survive the first 30 days.

Arrhythmias (Table S10)

In the Adult Cardiac Surgery Database arrhythmias included all forms of arrhythmia requiring treatment (such as resuscitation because of cardiac arrest or new onset atrium fibrillation or flutter that necessitates intervention (defibrillation or medication)). Spontaneous transient periods of atrial fibrillation without any consequence for the patient were not registered.

In the literature, the following clinical risk factors associated with arrhythmia following cardiac surgery are described: age, gender, hypertension, previous AF, previous cardiac surgery, congestive heart failure, COPD, right coronary artery disease, peripheral vascular disease, LV function, left ventricular hypertrophy, left atrial enlargement, electrocardiographic features, renal failure, moderate or severe aortic atherosclerosis, withdrawal beta-blocker or ACE-I, BSA, obesity and metabolic syndrome, diabetes, hypertension, aortic cross-clamp time, bicaval canulation, pulmonary vein venting, type of surgery, emergency surgery, need of perioperative intra-aortic balloon pump, CPB time, CPB inclusive of cardioplegic arrest, systemic hypothermia, respiratory compromise, red cell transfusion. [14-16]

Unfortunately, many of these variables were not available or there was a large proportion of missing values in the Adult Cardiac Surgery Database. The following variables were available and were included in a logistic regression model predicting arrhythmia after AVR: age, gender, previous cardiac surgery, COPD, peripheral vascular disease, LV function, renal failure, single vs. Concomitant other valve replacement,) concomitant CABG, aorta ascendens, aorta descendens, and aortic arch surgery, and emergency surgery. The tables below show the results of the logistic regression analyses of arrhythmias for patients that do and do not survive the first 30 days.

Myocardial infarction (Table S11)

MI are registered according to the definition used in the STS Adult Cardiac Surgery Database.[5] Possible predictors of MI after cardiac surgery in the literature are: age, gender, renal failure, diabetes, peripheral vascular disease, emergency surgery, redo surgery, LV dysfunction, perioperative MI, and concomitant CABG.[17, 18] The tables below show the results of the logistic regression analyses of myocardial infarctions for patients that do and do not survive the first 30 days.

Table S5. Comparison of patient- and procedure related risk factors, peri- and postoperative complications, and early mortality in complete cases and cases with at least one missing value in the Adult Cardiac Surgery Database (AVR).

	**Complete cases**		**Cases with ≥1 missing value**		
	n	mean/proportion	N	mean/proportion	p-value
Total (n)	32,345	90.5%	3,387	9.5%	
**Patient related risk factors**					
Age		68.6		67.0	0.000
Gender, male	20,122	62.21%	2,107	62.23%	1.000
Previous cardiac surgery	3,230	9.99%	31	9.39%	0.791
Preoperative serum creatinine level > 200 µmol/l	615	1.90%	88	2.89%	0.000
LV function					0.001
LVEF >50%	25,264	78.11%	2,627	79.39%	
LVEF 30-50%	5,707	17.64%	585	17.68%	
LVEF <30%	1,374	4.25%	97	2.93%	
COPD	4,481	13.85%	449	13.46%	0.551
Peripheral vascular disease	3,637	11.24%	329	9.87%	0.017
Neurological dysfunction	978	3.02%	86	2.57%	0.162
**Procedure related risk factors**					
Emergency procedure	1,090	3.37%	119	3.56%	0.589
Concomitant CABG	11,745	36.31%	1,097	32.39%	0.000
Concomitant aortic root procedure	357	1.10%	13	0.38%	0.000
Concomitant Bentall procedure	1,336	4.13%	48	1.42%	0.000
Concomitant aorta ascendens procedure	446	1.38%	8	0.24%	0.000
**Early mortality**	1,137	3.52%	112	3.31%	0.562

Table S6. Variables included in the multiple imputation model of the Adult Cardiac Surgery Database.

**Patient characteristics**	**Intervention characteristics**
Age	Year
Gender	Circular arrest
Creatinine level serum > 200 µmol/l	Type of aortic valve prosthesis
LV function	Extracorporeal circulation
- LVEF > 50%	Emergency surgery
- LVEF 30-50%	
- LVEF <30%	**Concomitant procedures**
COPD	CABG
Peripheral vascular disease	Valve repair
Neurological dysfunction	Concomitant other valve replacement
Endocarditis	Aortic root procedure
Critical preoperative state	Bentall procedure
Instable angina pectoris	Aorta ascendens procedure
Recent MI	Aortic arch procedure
Pulmonary hypertension	Aorta descendens procedure
	Aorta surgery
**Previous procedures**	Rhythm surgery
Cardiac surgery	Other cardiac surgery
Valve replacement	
	**Postoperative outcome**
	Early mortality

COPD = chronic obstructive pulmonary disease. MI = myocardial infarction.

Table S7. Logistic regression model of early mortality after aortic valve replacement (AVR) with multiple imputed data and complete case analysis of the Adult Cardiac Surgery Database.

	Multiple imputed data (n=35,732)				Complete cases (n=32,345)			
	**Estimate (log odds)**	**Odds ratio (OR)**	**CI 2.5% OR**	**CI 97.5% OR**	**Estimate (log odds)**	**Odds ratio (OR)**	**CI 2.5% OR**	**CI 97.5% OR**
Intercept	-6.712	0.001	0.001	0.002	-6.581	0.001	0.001	0.002
**Preoperative risk factors**								
Age	0.037	1.038	1.031	1.045	0.035	1.036	1.029	1.043
Gender, male	-0.450	0.637	0.564	0.720	-0.452	0.636	0.560	0.723
Previous cardiac surgery	1.069	2.912	2.486	3.411	1.060	2.885	2.462	3.381
Preoperative serum creatinine level > 200 µmol/l	0.926	2.525	1.950	3.270	1.013	2.755	2.108	3.600
LV function (compared to LVEF >50%)								
LVEF 30-50%	0.450	1.569	1.368	1.799	0.433	1.542	1.335	1.780
LVEF <30%	0.848	2.335	1.879	2.900	0.832	2.297	1.835	2.876
COPD	0.557	1.746	1.513	2.016	0.515	1.673	1.437	1.948
Peripheral vascular disease	0.478	1.613	1.390	1.872	0.442	1.555	1.330	1.820
Neurological dysfunction	0.409	1.505	1.155	1.961	0.465	1.592	1.215	2.086
**Procedure related risk factors**								
Emergency procedure	1.919	6.814	5.694	8.154	1.893	6.640	5.503	8.012
Concomitant CABG	0.677	1.967	1.737	2.229	0.704	2.022	1.774	2.304
Concomitant aorta root procedure	0.514	1.672	1.036	2.699	0.482	1.619	0.991	2.646
Concomitant Bentall procedure	0.917	2.503	1.969	3.181	0.924	2.518	1.971	3.218
Concomitant aorta ascendens procedure	0.705	2.024	1.304	3.141	0.727	2.069	1.332	3.212

Table S8. Logistic regression of risk on stroke during the first 30 days in patients that survive or do not survive the first 30 days after the intervention.

	Stroke in patients that do <u>not</u> survive first 30 days (complete cases n=417)					Stroke in patients that survive first 30 days (complete cases n=13087)				
	**Estimate (log odds)**	**Odds ratio (OR)**	**CI 2.5% (OR)**	**CI 97.5% (OR)**	**p-value**	**Estimate (log odds)**	**Odds ratio (OR)**	**CI 2.5% (OR)**	**CI 97.5% (OR)**	**p-value**
Intercept	0.471	1.601	3.418	1.466	0.702	2.263	0.005	0.002	0.014	0.000
Age	-0.041	0.960	1.016	0.079	0.011	-0.049	1.021	1.008	1.035	0.002
Male	0.282	1.326	1.378	2.410	0.379	0.206	0.613	0.476	0.790	0.000
Previous CVA	0.133	1.142	1.622	1.316	0.784	0.029	1.665	1.174	2.361	0.004
Previous cardiac surgery	-0.267	0.765	1.545	0.541	0.539	-0.382	1.978	1.368	2.859	0.000
Preoperative serum creatinine level > 200 µmol/l	-1.289	0.276	2.983	0.307	0.238	-0.894	0.827	0.297	2.305	0.717
LV function: LVEF > 50%	1.207	3.345	1.506	19.107	0.003	-0.441	0.833	0.623	1.112	0.215
COPD	-0.566	0.568	1.557	0.278	0.201	0.568	1.119	0.792	1.580	0.524
PVD	-0.156	0.855	1.533	0.694	0.714	-1.366	1.236	0.865	1.766	0.244
Neurological dysfunction	-0.485	0.616	2.019	0.502	0.490	-0.716	1.659	0.928	2.964	0.088
Instable angina pectoris	1.660	5.257	2.176	8.456	0.033	-17.516	0.870	0.263	2.878	0.820
Pulmonary hypertension	-0.917	0.400	3.104	0.445	0.418	-0.568	0.528	0.192	1.451	0.216
Endocarditis	-0.239	0.787	1.628	0.612	0.624	-0.162	0.534	0.263	1.087	0.084
Emergency surgery	-0.022	0.978	1.484	0.945	0.955	-0.465	3.811	2.185	6.648	0.000
Concomitant other valve replacement	-0.847	0.429	1.800	0.237	0.150	-0.171	1.146	0.594	2.208	0.684
Concomitant CABG	-0.489	0.613	1.395	0.230	0.142	0.070	1.505	1.157	1.958	0.002
Concomitant aorta ascendens procedure	0.480	1.617	1.783	2.295	0.406	1.926	1.530	0.823	2.842	0.179
Apparent AUC	0.724					0.662				
Optimism	0.073					0.027				
Bootstrapped AUC	0.651					0.636				

Table S9. Logistic regression of early risk on renal failure during the first 30 days in patients that survive or do not survive the first 30 days after the intervention.

	Renal failure in patients that do not survive first 30 days (complete cases n=622)					Renal failure in patients that survive first 30 days (complete cases n=17191)				
	Estimate (log odds)	Odds ratio (OR)	CI 2.5% (OR)	CI 97.5% (OR)	p-value	Estimate (log odds)	Odds ratio (OR)	CI 2.5% (OR)	CI 97.5% (OR)	p-value
Intercept	-2.507	0.081	0.014	0.485	0.006	-5.683	0.003	0.002	0.007	0.000
Age	0.011	1.011	0.987	1.035	0.375	0.018	1.018	1.008	1.028	0.000
Male	-0.092	0.912	0.593	1.402	0.674	0.229	1.258	1.006	1.573	0.045
Previous cardiac surgery	-0.183	0.833	0.482	1.441	0.514	1.059	2.884	2.254	3.690	0.000
Preoperative serum creatinine level > 200 µmol/l	0.459	1.583	0.678	3.694	0.288	1.968	7.154	5.109	10.019	0.000
LV function (compared to LVEF >50%)										
LVEF 30-50%	0.175	1.191	0.736	1.926	0.477	0.312	1.367	1.079	1.731	0.010
LVEF <30%	-0.519	0.595	0.254	1.396	0.233	0.785	2.193	1.490	3.228	0.000
COPD	0.197	1.218	0.730	2.031	0.451	0.377	1.458	1.127	1.888	0.004
PVD	0.124	1.132	0.674	1.902	0.639	0.530	1.699	1.312	2.200	0.000
Emergency surgery	0.444	1.559	0.904	2.689	0.110	1.477	4.380	3.144	6.100	0.000
Concomitant CABG	0.154	1.166	0.747	1.822	0.499	0.001	1.001	0.802	1.250	0.990
Apparent AUC	0.583					0.723				
Optimism	0.070					0.012				
Bootstrapped AUC	0.513					0.711				

Table S10. Logistic regression of early risk on arrhythmias during the first 30 days in patients that survive or do not survive the first 30 days after the intervention.

	Arrhythmias in patients that do not survive first 30 days (complete cases n=623)					Arrhythmias in patients that survive first 30 days (complete cases n=17192)				
	Estimate (log odds)	Odds ratio (OR)	CI 2.5% (OR)	CI 97.5% (OR)	p-value	Estimate (log odds)	Odds ratio (OR)	CI 2.5% (OR)	CI 97.5% (OR)	p-value
Intercept	-1.953	0.142	0.030	0.677	0.014	-2.235	0.107	0.085	0.135	0.000
Age	0.016	1.016	0.995	1.038	0.125	0.024	1.024	1.021	1.028	0.000
Male	-0.568	0.566	0.392	0.819	0.003	0.050	1.051	0.984	1.124	0.141
Previous cardiac surgery	-0.075	0.927	0.580	1.482	0.752	-0.066	0.936	0.838	1.046	0.244
Preoperative serum creatinine level > 200 µmol/l	0.917	2.501	1.194	5.242	0.015	0.013	1.013	0.793	1.294	0.919
LV function (compared to LVEF >50%)										
LVEF 30-50%	-0.027	0.973	0.632	1.498	0.903	0.062	1.064	0.980	1.156	0.140
LVEF <30%	0.213	1.237	0.656	2.332	0.511	0.144	1.154	0.976	1.366	0.094
COPD	0.217	1.243	0.798	1.936	0.337	0.062	1.064	0.970	1.166	0.190
PVD	-0.211	0.810	0.500	1.310	0.390	-0.010	0.990	0.894	1.097	0.851
Emergency surgery	-0.183	0.833	0.484	1.433	0.509	0.014	1.014	0.831	1.237	0.891
Concomitant other valve replacement	-0.067	0.935	0.545	1.606	0.808	0.548	1.729	1.466	2.039	0.000
Concomitant CABG	0.247	1.280	0.868	1.887	0.213	0.015	1.015	0.948	1.087	0.667
Concomitant aorta ascendens procedure	0.077	1.080	0.391	2.988	0.882	0.197	1.217	0.989	1.497	0.063
Concomitant aorta descendens procedure	1.888	6.603	0.826	52.787	0.075	0.107	1.113	0.466	2.654	0.810
Concomitant aortic arch procedure	-0.889	0.411	0.150	1.125	0.084	-0.205	0.814	0.657	1.010	0.061
Apparent AUC	0.641					0.573				
Optimism	0.046					0.004				
Bootstrapped AUC	0.595					0.569				

Table S11. Logistic regression of early risk on MI during the first 30 days in patients that survive or do not survive the first 30 days after the intervention.

	MI in patients that do not survive first 30 days (complete cases n=505)					MI in patients that survive first 30 days (complete cases n=13,831)				
	**Estimate (log odds)**	**Odds ratio (OR)**	**CI 2.5% (OR)**	**CI 97.5% (OR)**	**p-value**	**Estimate (log odds)**	**Odds ratio (OR)**	**CI 2.5% (OR)**	**CI 97.5% (OR)**	**p-value**
Intercept	0.354	1.425	0.152	13.343	0.756	-4.315	0.013	0.005	0.039	0.000
Age	-0.036	0.965	0.935	0.995	0.023	-0.010	0.990	0.975	1.005	0.185
Male	-0.584	0.558	0.308	1.010	0.054	-0.223	0.800	0.577	1.109	0.181
Previous cardiac surgery	-0.753	0.471	0.158	1.402	0.176	0.631	1.880	1.117	3.164	0.018
LV function (compared to LVEF >50%)						-0.160	0.852	0.563	1.291	0.451
LVEF 30-50%	-0.075	0.927	0.465	1.848	0.830	-0.330	0.719	0.291	1.777	0.475
LVEF <30%	-0.678	0.508	0.143	1.801	0.294	0.328	1.388	0.920	2.096	0.118
PVD	-0.259	0.772	0.355	1.680	0.514	1.086	2.962	1.515	5.792	0.002
Emergency surgery	-0.486	0.615	0.248	1.527	0.295	-0.601	0.548	0.158	1.903	0.344
Concomitant other valve replacement	-0.107	0.899	0.152	5.324	0.906	1.301	3.674	2.602	5.188	0.000
Concomitant CABG	1.100	3.003	1.535	5.875	0.001	-4.315	0.013	0.005	0.039	0.000
Apparent AUC	0.684					0.677				
Optimism	0.061					0.008				
Bootstrapped AUC	0.622					0.669				

Late events

The occurrence of late events after SAVR was based on our previously published systematic review and meta-analysis. For more details regarding the methods and results of this study we refer to the publication in the Journal of Thoracic and Cardiovascular Surgery.[8]

2.3.2 Transcatheter aortic valve implantation (TAVI) in elderly

Early mortality and events

The clinical outcomes after TAVI are derived from a systematic review performed by Gargiulo et al. (2016).[19] This study included five randomized trials and 31 observational matched studies comparing outcomes after TAVI or SAVR. Gargiulo et al. [19] only report odds ratios of clinical outcomes after TAVI compared to SAVR instead of the mortality and event risks and rates we needed as input for our patient level simulation model. Therefore, we have pooled the extracted data reported in Gargiulo et al. [19] with the use of the inverse variance method in a random-effects model, on a logarithmic scale, as the Shapiro–Wilk test revealed a significantly skewed distribution among the included studies in the outcome measures. In total 7,726 TAVI patients from 36 studies were included in the meta-analysis. Their mean age was 80.9 years, 50.3% were males, and the mean STS score was 6.7%.[19] The risks on mortality and events during the first 30 days after TAVI are reported in Table 1 in the main manuscript. We assume these risks are equal for all patients, whether they die within 30 days or not. The other early event risks (i.e. prosthetic valve thrombosis and endocarditis) were assumed to be zero for all patients.

Late events

The rates of valve-related events after TAVI are derived from the systematic review of Gargiulo et al. on outcomes after TAVI.[19] The long term event occurrence was not reported in the meta-analyses of Gargiulo et al. Therefore we extracted and pooled the relevant data from the included studies ourselves (Table 1 in main manuscript).

Prosthetic valve dysfunction (besides early aortic regurgitation due to paravalvular leak) is not often reported in the currently available literature of outcomes after TAVI. Sokoloff et al. reported a Kaplan-Meier curve on freedom of SVD with a maximum follow-up of ten years. This Kaplan-Meier curves was digitized and an estimate of the individual patient time-to-event data was then extrapolated from the digitized curve coordinates, assuming a constant rate of censorship between each time point at which the number of patients at risk were specified.[20] The occurrence rate of SVD after AVR with bioprostheses and TAVI was modelled by fitting a gompertz or lognormal distribution to our pooled time-to-event data, respectivelly, showing an increasing occurrence rate of SVD over time. These distributions had the best fit according to visual comparison, log-likelihood and/or Akaike information criterion (Table S12).

Table S12. Goodness of fit statistics of SVD after SAVR and TAVI

	**SVD after SAVR**		**SVD after TAVI**	
**Distribution**	**Log likelihood**	**AIC**	**Log likelihood**	**AIC**
Exponential	-377.43	756.87	-24.10	50.20
Weibull	-368.69	741.37	-20.01	44.01
Gamma	-369.40	742.80	-19.73	43.47
Gompertz	**-366.21**	**736.42**	-21.12	46.24
Generalized gamma	-367.71	741.43	**-19.24**	44.49
Log normal	-376.42	756.83	-19.54	**43.08**
Log logistic	-369.08	742.18	-19.91	43.81

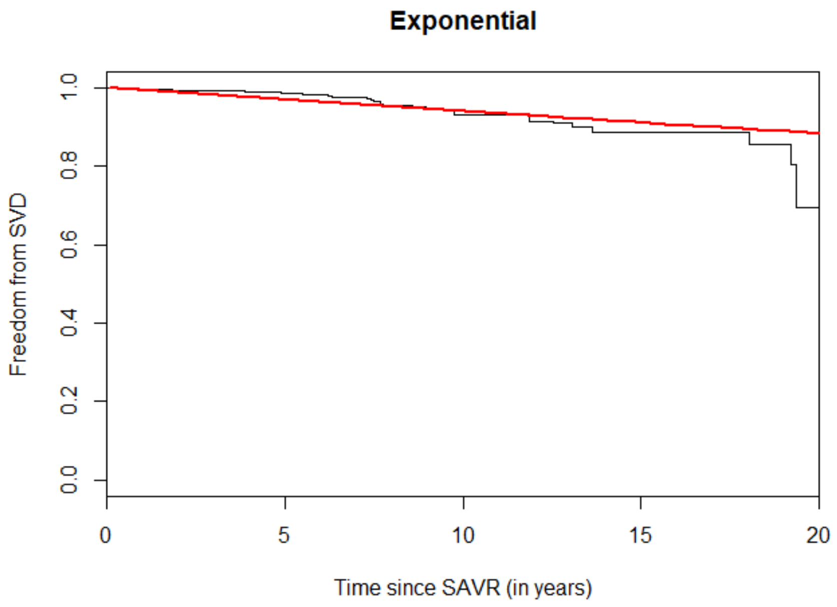

Figure S2. Visual fit of exponential distribution to freedom from SVD after SAVR.

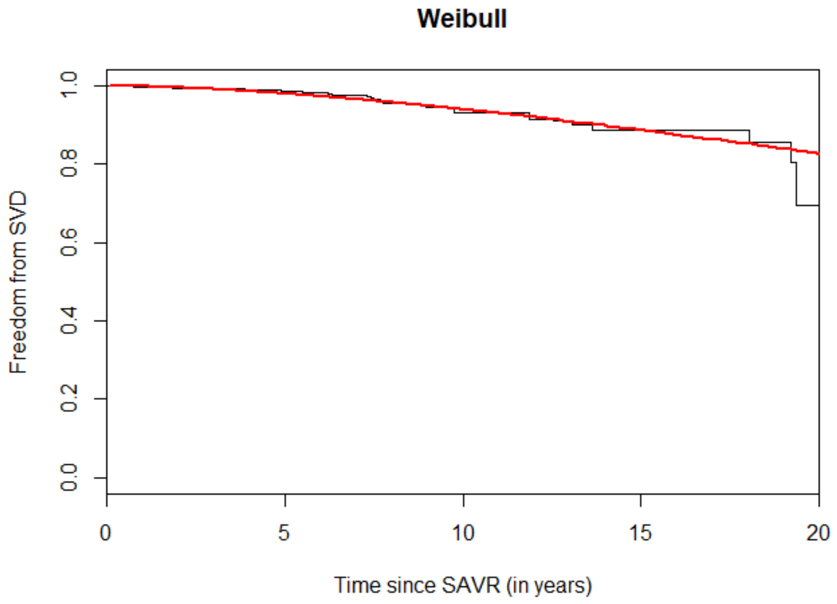

Figure S3. Visual fit of Weibull distribution to freedom from SVD after SAVR.

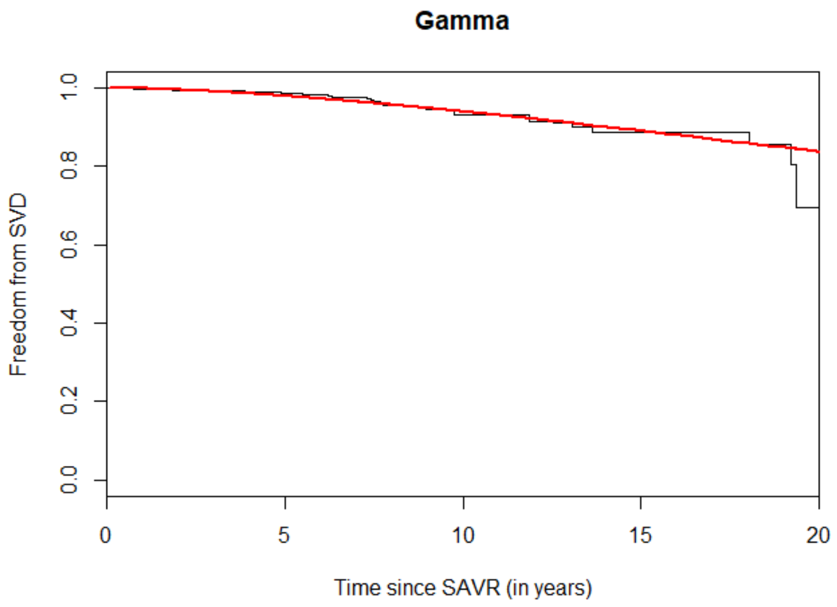

Figure S4. Visual fit of gamma distribution to freedom from SVD after SAVR.

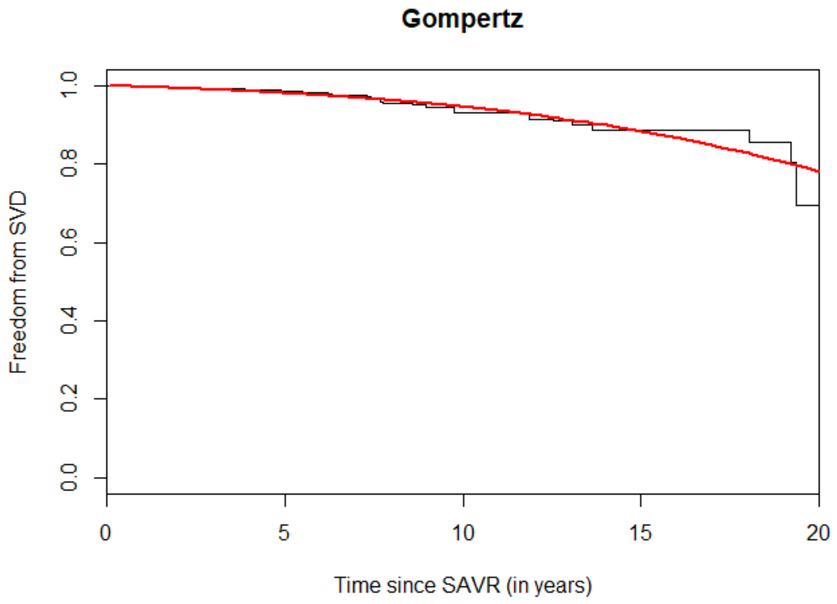

Figure S5. Visual fit of Gompertz distribution to freedom from SVD after SAVR.

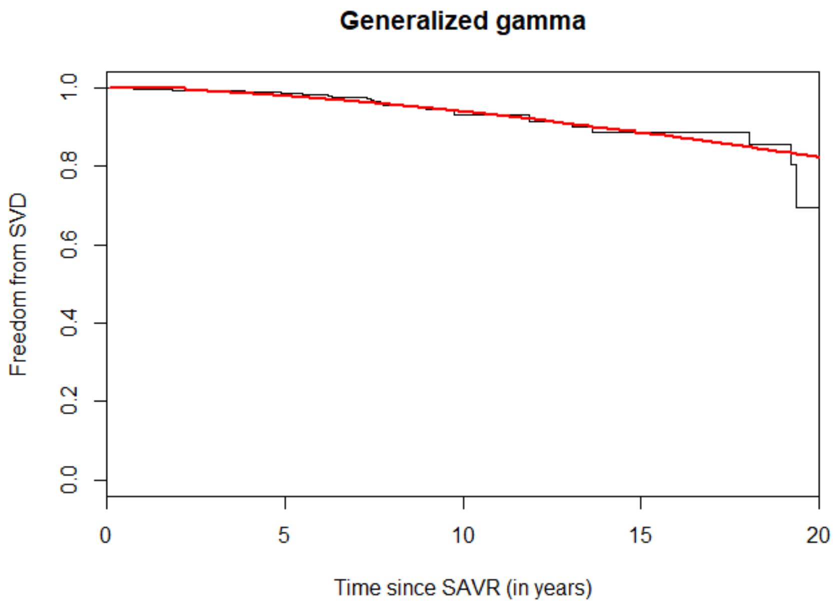

Figure S6. Visual fit of generalized gamma distribution to freedom from SVD after SAVR.

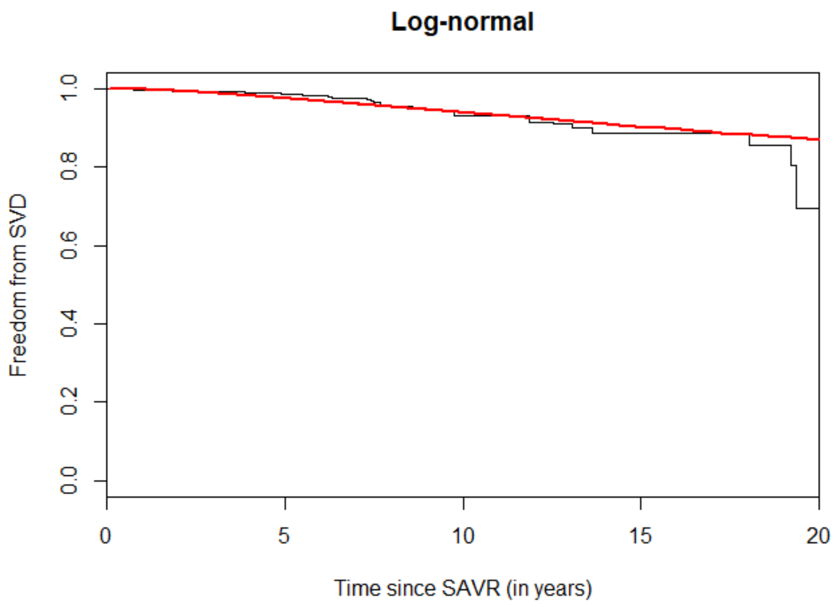

Figure S7. Visual fit of log-normal distribution to freedom from SVD after SAVR.

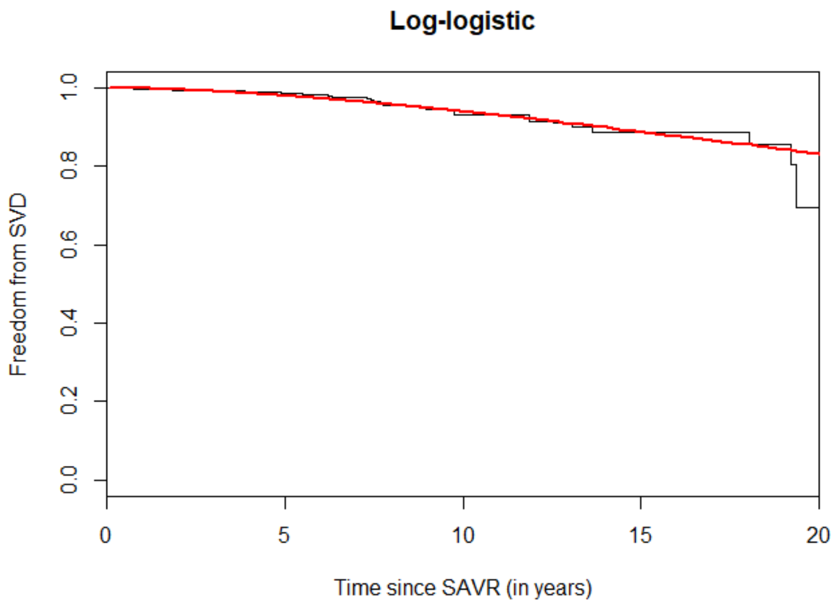

Figure S8. Visual fit of log-logistic distribution to freedom from SVD after SAVR.

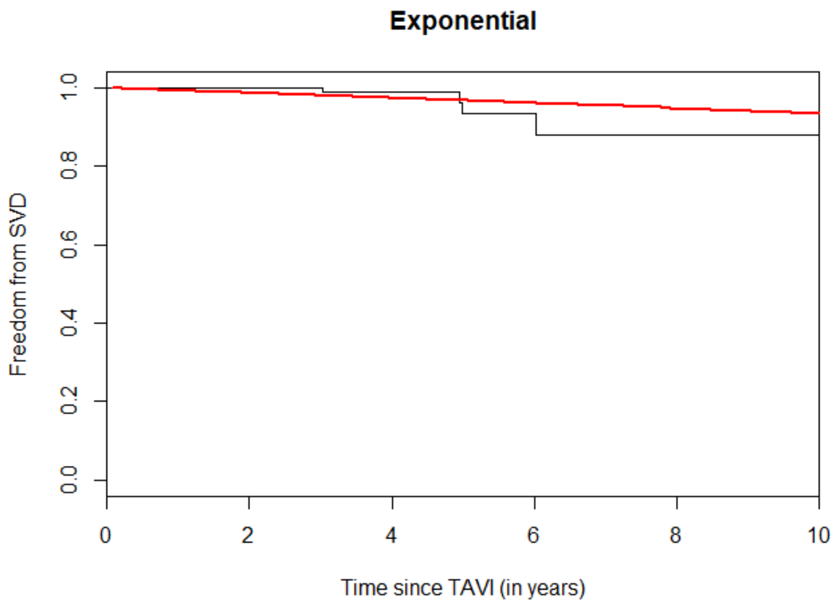

Figure S9. Visual fit of exponential distribution to freedom from SVD after TAVI.

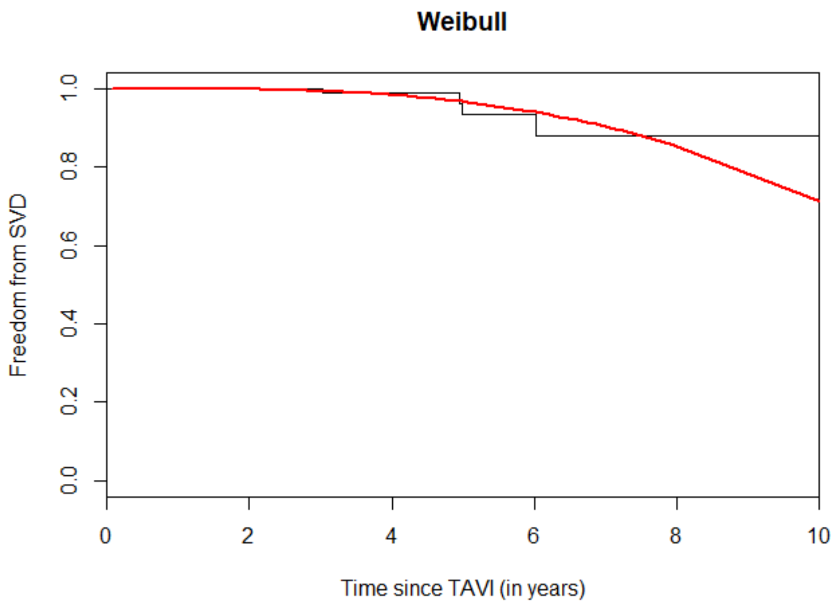

Figure S10. Visual fit of Weibull distribution to freedom from SVD after TAVI.

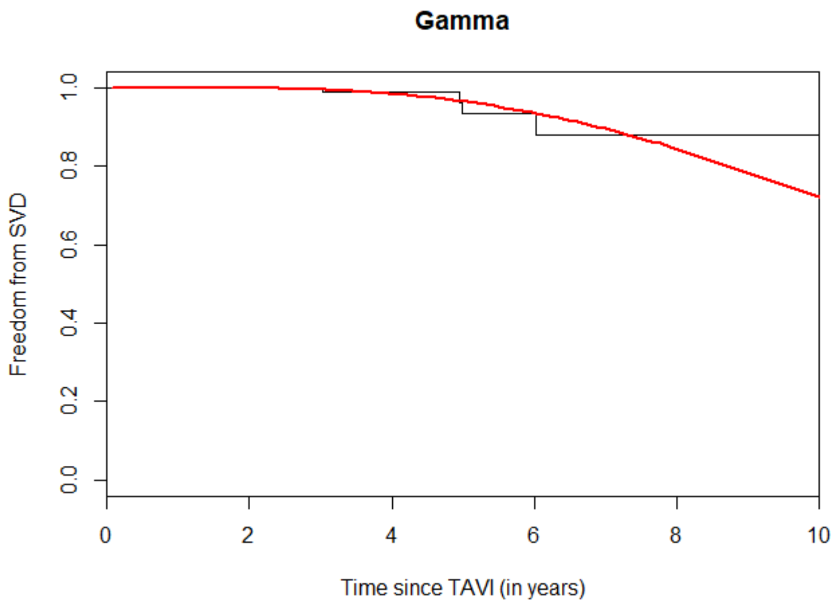

Figure S11. Visual fit of gamma distribution to freedom from SVD after TAVI.

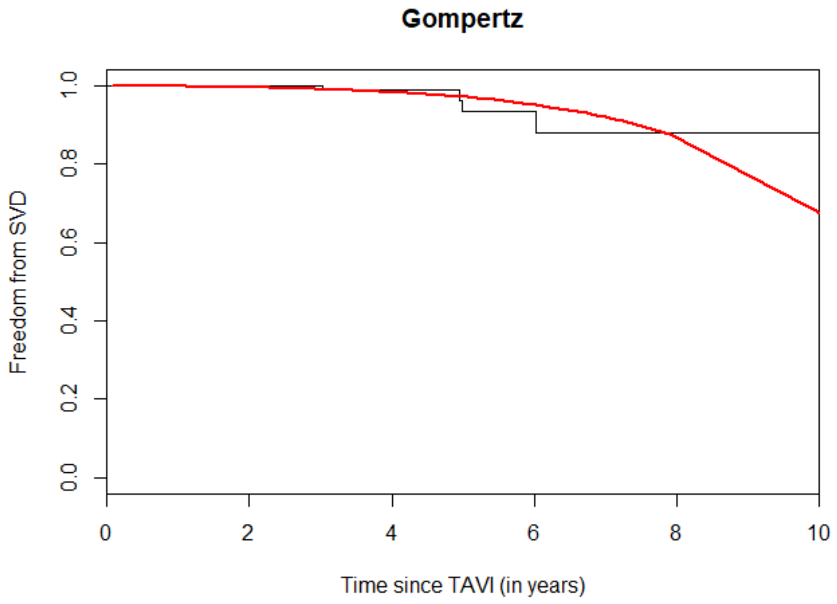

Figure S12. Visual fit of Gompertz distribution to freedom from SVD after TAVI.

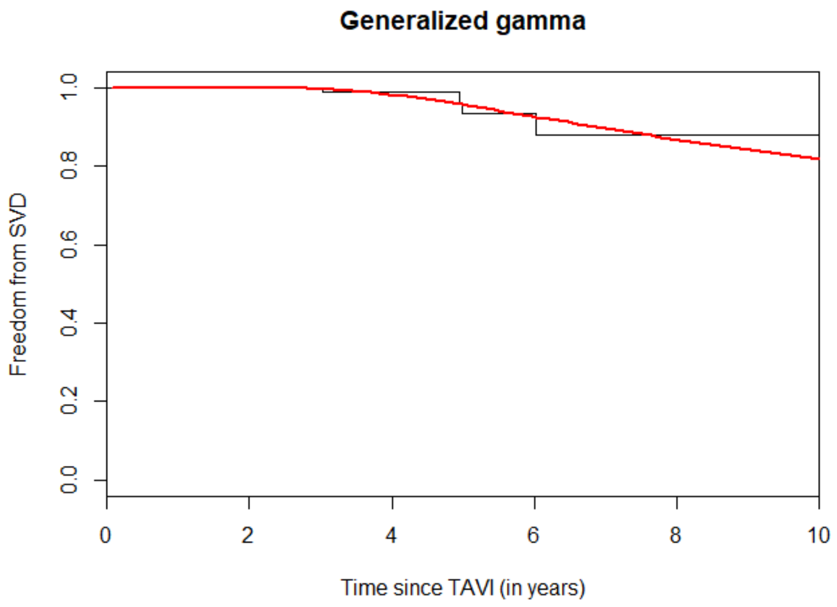

Figure S13. Visual fit of generalized gamma distribution to freedom from SVD after TAVI.

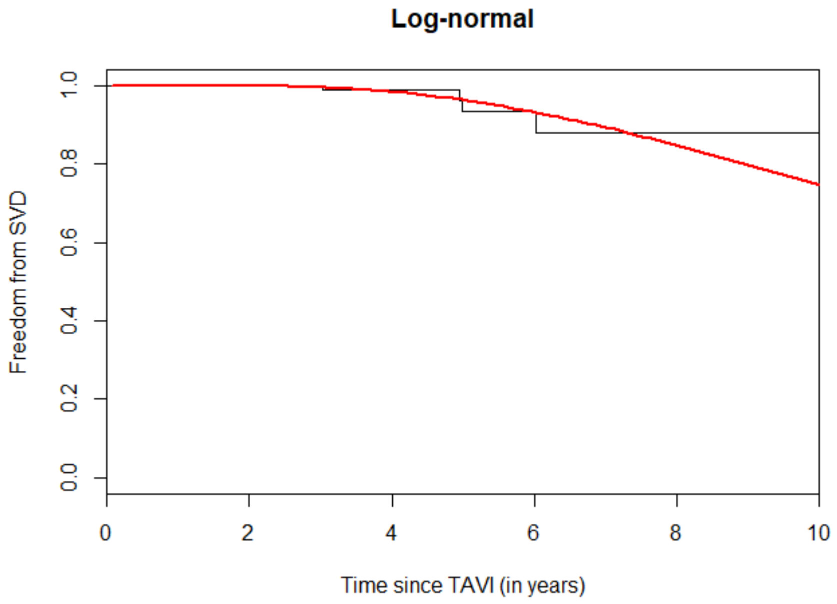

Figure S14. Visual fit of log-normal distribution to freedom from SVD after TAVI.

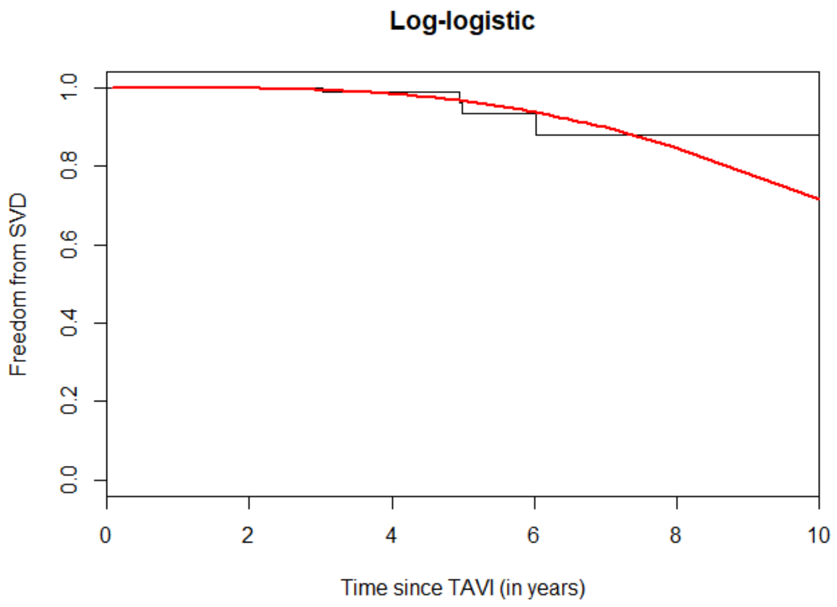

Figure S15. Visual fit of log-normal distribution to freedom from SVD after TAVI.

The occurrence of **valve thrombosis** (VT) was not reported in any of the studies included in the systematic review of Gargiulo et al. Therefore the linearized occurrence rate of VT was based on the Bern TAVI Registry (0.69%/patient-year).[21] Blackstone & Kirklin have shown that valve thrombosis mainly occurs during the first year after surgical mechanical aortic valve replacement and deteriorates to almost zero after six years.[22] The higher occurrence in the early phase may be caused by suboptimal anticoagulation treatment in the first post-intervention period. Since, the mean follow-up of the Bern TAVI Registry was only one year, it is likely that the occurrence rate of valve thrombosis after TAVI found in this study will not remain constant but will reduce over time. Therefore we recalculated the linearized occurrence rate, assuming that it will be zero from year 7 onwards. The adjusted linearized occurrence rate of VT is then 0.24%/patient-year.

The pooled linearized occurrence rate of **endocarditis** (0.54%/patient-year) was based on studies with a mean follow-up of 1.9 years, since this rate is comparable to the rate of endocarditis after SAVR, we assume this will remain constant over time.

The linearized occurrence rates of **stroke** (2.03%/patient-year) and **bleeding** (3.34%/patient-year) were relatively high after TAVI. However, this is probably caused by the short mean follow-up duration of the studies that reported long term stroke and bleeding (1.8 and 2.5 years, respectively), since bleedings and strokes mainly occur in the earliest period after TAVI.[23] In patients after SAVR we have seen that (probably due to higher use of anticoagulation) the occurrence of strokes is lower (HR: 0.7) and of bleeding is higher (HR: 5.3) than in the age and sex matched general population. The late occurrence of stroke and bleedings was comparable between SAVR and TAVI in two large randomized controlled trials.[24, 25] Therefore we applied the same hazard ratio of strokes of SAVR patients compared to the general population to the general population with comparable age and sex as the TAVI population. Therefore we applied the hazard ratios determined for SAVR patients to the occurrence of stroke and bleeding in the age and sex matched general population for TAVI resulting in a linearized occurrence rate of strokes of 0.955%/patient-year and bleeding of 0.954%/patient-year.

Supplement 5 – Cost input parameters

Table S13. Multilevel generalized linear model for the other annual healthcare costs after SVR in post-intervention years 1 through 4.

**Other healthcare costs**	**Adults (n=17,553)**		
**Parameter**	**β**	**95% CI**	**P-value**
**Intercept**	11,662	10,315-13,009	<.0001
**Time** (compared to year 1 excluding intervention costs)			
Year 2	-3,461	-4,308--2,614	<.0001
Year 3	-2,372	-3,920--0,823	0.003
Year 4	-0,243	-2,252-1,766	0.812
**Death**	3,845	2,672-5,018	<.0001
**Age at intervention** (compared to elderly)			
Young adults	-1,070	-2,123--0,017	0.046
Middle aged	-2,373	-3,242--1,505	<.0001
**Male**	-0,940	-1,694--0,185	0.015
**Co-morbidity** (compared to no co-morbidity)			
COPD, DM, kidney disease and/or HF	6,357	5,216-7,498	<.0001
Hypertension	1,378	0,207-2,548	0.021
Other co-morbidities	1,964	0,289-3,639	0.022
**SES**[1] (compared to highest SES: 71-100)			
0-20	1,235	0,174-2,295	0.023
21-40	0,320	-0,741-1,381	0.554
41-70	0,833	-0,125-1,791	0.088
**Complications**			
AF	747	-0,485-1,979	0.235
AKI	8,178	5,371-10,985	<.0001
Stroke	4,506	3,038-5,974	<.0001
MI	5,677	2,005-9,350	0.002
PI	3,430	1,438-5,423	0.001

COPD = Chronic Obstructive Pulmonary Disease. DM = diabetes mellitus. HF = heart failure. SES = Socioeconomic status. AF = atrial fibrillation. AKI = acute kidney injury. TIA = transient ischemic attack. MI = myocardial infarction. PI = pacemaker implantation.
[1]Higher percentiles represent higher SES.

Table S14. Regression analyses of productivity costs of unpaid work in SAVR patients

A. Probability unpaid work	SAVR (n=625)			TAVI (n=213)		
	Coefficient	Odds ratio (95% CI)	p-value	Coefficient	Odds ratio (95% CI)	p-value
(Intercept)	-0.742	0.476 (0.165-1.375)	0.170	0.117	1.124 (0.030-42.508)	0.950
Age	0.003	1.003 (0.986-1.021)	0.720	-0.016	0.984 (0.941-1.028)	0.470
Male	0.117	1.125 (0.776-1.629)	0.535	0.672	1.959 (1.026-3.739)	0.041
Years since intervention	0.061	1.063 (0.969-1.166)	0.196	-0.035	0.966 (0.804-1.161)	0.712
Biological valve (compared to mechanical)	-0.057	0.945 (0.593-1.505)	0.812			
Concomitant CABG	0.269	1.308 (0.881-1.943)	0.182			
Multiple valve replacement	0.188	1.207 (0.692-2.105)	0.508			
**B. Probability less unpaid work**	**SAVR (n=257)**			**TAVI (n=65)[1]**		
	**Coefficient**	**Odds ratio (95% CI)**	**p-value**	**Coefficient**	**Odds ratio (95% CI)**	**p-value**
(Intercept)	2.609	13.582 (1.745-105.694)	0.013	-2.657	0.070 (0.030-0.164)	0.000
Age	-0.050	0.952 (0.919-0.985)	0.005			
Male	-0.182	0.834 (0.419-1.661)	0.605	-0.260	0.771 (0.305-1.947)	0.582
Years since intervention	-0.253	0.776 (0.648-0.930)	0.006	0.183	1.200 (0.961-1.500)	0.108
Biological valve (compared to mechanical)	0.700	2.014 (0.832-4.872)	0.121			
Concomitant CABG	-0.504	0.604 (0.278-1.316)	0.205			
Multiple valve replacement	-0.360	0.698 (0.219-2.221)	0.542			
**C. Estimated productivity costs unpaid work last four weeks**	**SAVR (n=57)**			**TAVI (n=16)[2]**		
	**Coefficient**	**Exponentiated coefficient† (95% CI)**	**p-value**	**Mean productivity costs of unpaid work in all patients with less unpaid work**		
(Intercept)	6.272	529.373 (136.433-2054.015)	0.000	€648±€797		
Age	-0.003	0.997 (0.974-1.021)	0.807			
Male	0.068	1.070 (0.614-1.864)	0.812			
Years since intervention	-0.104	0.901 (0.781-1.040)	0.161			
Biological valve (compared to mechanical)	0.763	2.145 (1.084-4.242)	0.033			
Concomitant CABG	-0.535	0.586 (0.295-1.163)	0.133			
Multiple valve replacement	1.223	3.398 (1.284-8.995)	0.017			

†The exponentiated coefficient is the factor by which the arithmetic mean outcome on the original scale is multiplied. N.B. The results should be interpreted as follows, for example for sex: males are more likely to have unpaid work than females (logistic regression model 1), are less likely to be unable to perform unpaid work (logistic regression model 2) and when all other variables are equal, the mean estimated productivity costs of males is almost equal to females(GLM). [1]Age excluded because number of events (n=21) was too small for three predictors. [2]The productivity costs of patients who performed less unpaid work after TAVI was only reported for 16 of the 21 patients with less unpaid work. Therefore we apply the average productivity costs of unpaid work in these patients to all patients instead of using a GLM.

Table S15. Regression analyses of informal care use and costs

	SAVR (n=625)			TAVI (n=248)		
**A. Probability of using informal care**	**Coefficient**	**Odds ratio (95% CI)**	**p-value**	**Coefficient**	**Odds ratio (95% CI)**	**p-value**
Intercept	-1.179	0.308 (0.071-1.334)	0.115	0.402	1.495 (0.062-36.225)	0.805
Age	0.012	1.012 (0.987-1.038)	0.334	-0.004	0.996 (0.958-1.035)	0.847
Male	-0.678	0.508 (0.323-0.797)	0.003	-0.767	0.464 (0.270-0.798)	0.005
Years since intervention	-0.318	0.727 (0.639-0.828)	0.000	-0.141	0.868 (0.735-1.027)	0.099
Biological valve (compared to mechanical)	-0.111	0.895 (0.476-1.684)	0.731			
Concomitant CABG	-0.134	0.874 (0.515-1.484)	0.619			
Multiple valve replacement	0.442	1.556 (0.798-3.032)	0.194			
	**SAVR (n=97)**			**TAVI (n=86)**		
**B. Estimated informal care costs/week (gamma family, inverse link)**	**Coefficient**	**95% CI**	**p-value**	**Coefficient**	**95% CI**	**p-value**
Intercept	0.002	-0.002-0.007	0.326	0.003	-0.004-0.010	0.383
Age	0.000	0.000-0.000	0.336	0.000	0.000-0.000	0.488
Male	-0.001	-0.002-0.001	0.508	-0.003	-0.004--0.001	0.002
Years since intervention	0.000	0.000-0.000	0.897	0.000	0.000-0.000	0.902
Biological valve (compared to mechanical)	-0.002	-0.004-0.000	0.098			
Concomitant CABG	0.001	-0.001-0.003	0.562			
Multiple valve replacement	-0.001	-0.003-0.001	0.177			

CABG: coronary artery bypass grafting. The results should be interpreted as follows, for example in the models for SAVR patients: males are less likely to use informal care than females (logistic regression model) and when all other variables are equal, the mean estimated informal care costs of males is 0.001 times (i.e. 0.1%) lower than for females (GLM).

Table S16. Length of hospital stay after events.

Event	Length of hospital stay (LOS)
Bleeding	2 days [26]
Prosthetic valve dysfunction without re-intervention	8.67 days [27]
Valve thrombosis	10 days
Endocarditis	6 weeks

Supplement 6 – Probabilistic sensitivity analysis

To estimate the numbers of patients and simulations required in our probabilistic sensitivity analyses (PSA), we used the approach described in O'Hagan et al. as recommended by the NICE DSU guidelines on patient-level modelling.[28] In this approach the number of PSA runs (outer loop = N) and patients per PSA run (inner loop = n) needed to achieve accurate cost-effectiveness estimates while keeping the number of runs as small as possible can be estimated. The cost-effectiveness measure used in this estimation was the number of undiscounted QALYs.

The box below showed the approximations presented by O'Hagan et al. that we used. According to O'Hagan et al. the approximations are sufficiently accurate when k is at least 25 and c is less than or equal to 0.2.

M = $8k/c^2 = N*n$
n = $1 + k$

N = number of PSA runs (outer loop)
n = number of patients per PSA run (inner loop)
k = $\frac{\text{patient-level variance}}{\text{parameter variance}}$
c = coefficient of variance = $\frac{\text{SD parameter}}{\text{mean of parameter}}$

The patient-level variance was 27.37, estimated with a deterministic run of 25.000 patients. The parameter variance was 0.30, which was the mean of 500 model runs each of them based on 100 patients. Therefore, k was 27.37 / 0.30 = 92. Based on the formulas described above the number of patients per PSA run would be 92 + 1 = 93 (after rounding up). Assuming a c of 0.2, M = 18,679 and the number of PSA runs would be 200 (after rounding up). However, the choice of c = 0.2 was arbitrary and based on the minimum accuracy requirement and there is no generally accepted threshold value for c in the literature. Therefore, we chose to run the 500 PSA runs including 500 patients each, translating to a value for c of approximately 0.12 (i.e. almost twice more accurate).

Supplement 7 – Additional results

Table S17. SVD free life expectancy in various scenarios.

SVD free life expectancy (median)	SAVR	TAVI
**Current valve prostheses**	9.4	4.6
- Subgroup patients aged 70-80 years	10.4	6.9
- Subgroup patients aged >80 years	6.4	3.7
**Improved durability of TEHV**		
No prosthetic valve dysfunction events	lifetime	lifetime
75% less prosthetic valve dysfunction events	9.9	4.7
50% less prosthetic valve dysfunction events	9.9	4.7
25% less prosthetic valve dysfunction events	9.6	4.7
**Perfect TEHV (no prosthetic valve related events)**	lifetime	lifetime
**Improved TEHV (50% less prosthetic valve related events)**	10.0	4.7
- Subgroup patients aged 70-80 years	11.0	7.7
- Subgroup patients aged >80 years	6.6	3.8
**Decreased durability (50% more events) but improvements in thrombogenicity and infection resistance (50% less events)**	9.0	4.5

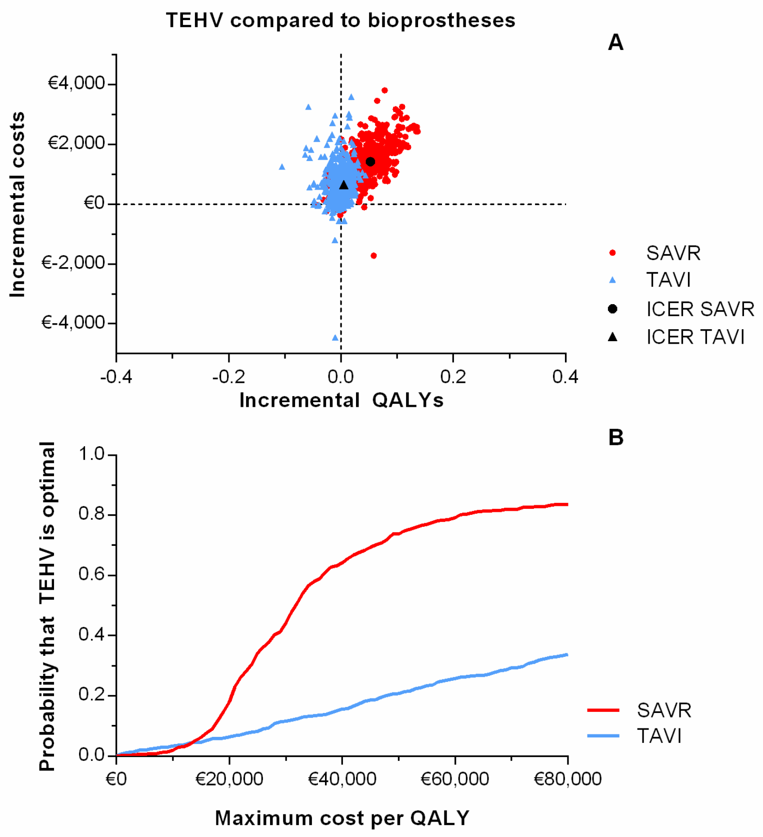

Figure S16. Probabilistic sensitivity analyses outcomes of surgical (SAVR) and transcatheter (TAVI) aortic valve implantation with TEHV (partially improved performance) compared to bioprostheses. A: Cost-effectiveness plane. B: Cost-effectiveness acceptability curve (CEAC)

Table S18. Cumulative cost savings per year in the first 10 years after introduction of TEHV with varying substitution rates

	SAVR (n=1,931/year)				TAVI (n=809/year)				TAVI (n=3,745/year)			
**Substitution rate TEHV**	25%	50%	75%	100%	25%	50%	75%	100%	25%	50%	75%	100%
**Years**												
1	57,124	114,247	171,371	228,495	7,606	15,212	22,818	30,424	35,210	70,419	105,629	140,839
2	192,446	384,891	577,337	769,782	25,012	50,024	75,035	100,047	115,784	231,568	347,352	463,136
3	389,685	779,369	1,169,054	1,558,738	51,834	103,667	155,501	207,335	239,947	479,894	719,840	959,787
4	628,621	1,257,242	1,885,863	2,514,484	91,706	183,413	275,119	366,825	424,524	849,049	1,273,573	1,698,098
5	910,997	1,821,995	2,732,992	3,643,989	148,536	297,071	445,607	594,143	687,597	1,375,195	2,062,792	2,750,389
6	1,224,539	2,449,078	3,673,616	4,898,155	226,671	453,343	680,014	906,686	1,049,301	2,098,602	3,147,902	4,197,203
7	1,569,697	3,139,393	4,709,090	6,278,786	326,901	653,802	980,703	1,307,604	1,513,281	3,026,561	4,539,842	6,053,123
8	1,953,514	3,907,028	5,860,543	7,814,057	439,586	879,171	1,318,757	1,758,343	2,034,918	4,069,835	6,104,753	8,139,671
9	2,364,815	4,729,630	7,094,444	9,459,259	561,726	1,123,452	1,685,177	2,246,903	2,600,325	5,200,650	7,800,975	10,401,300
10	2,809,036	5,618,071	8,427,107	11,236,142	692,159	1,384,317	2,076,476	2,768,635	3,204,121	6,408,243	9,612,364	12,816,485

Costs in Euros.

Supplement 8 – Internal validation

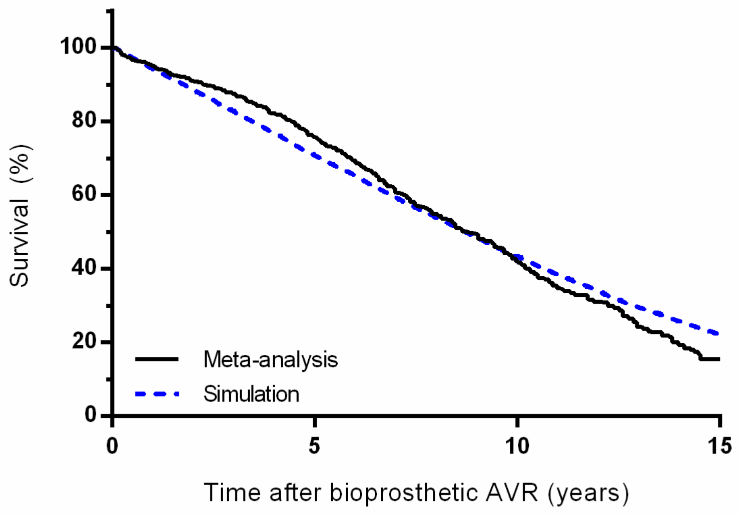

Figure S17. Internal validation of survival after SAVR with bioprostheses.

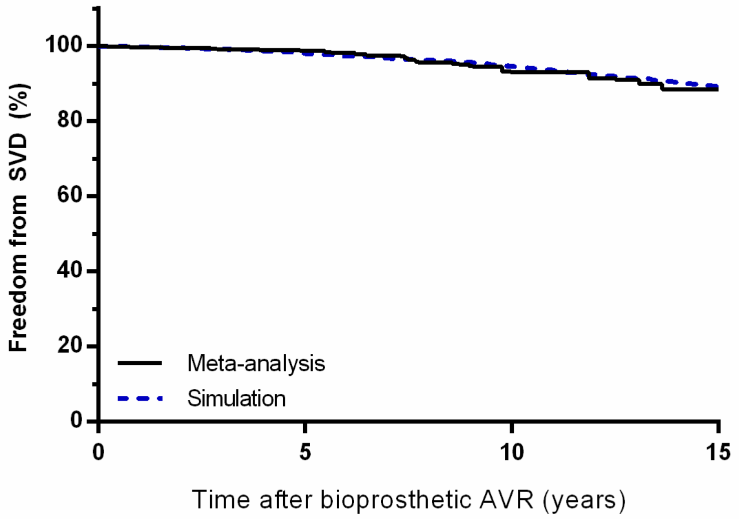

Figure S18. Internal validation of freedom from structural valve deterioration (SVD) after SAVR with bioprostheses.

Supplement 9 – External validation

Table S19. Mean age and proportion of patients with concomitant CABG in age and sex subgroups in the simulation model and observed data from the Providence Health System.

	Mean age (years)		Concomitant CABG (%)	
**Subgroups by age and sex**	**Simulation**	**Observed**	**Simulation**	**Observed**
70-80 years - males	74.9	75.1	49	56
70-80 years - females	75.3	75.4	35	39
>80 years - males	82.2	84.2	53	63
>80 years - females	82.4	84.3	39	48

CABG: coronary artery bypass grafting.

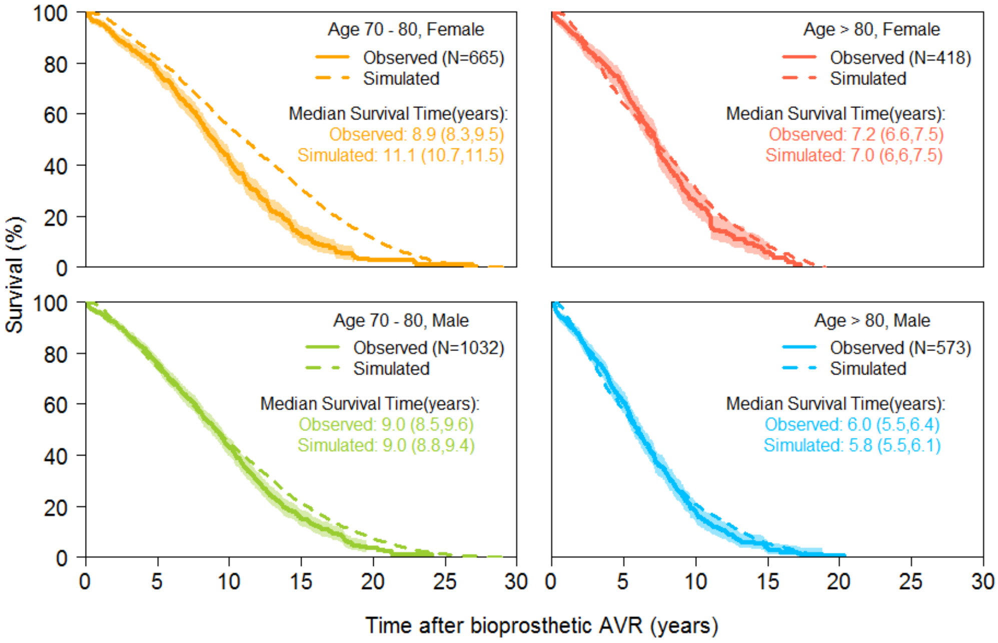

Figure S19. External validation of survival outcomes. Comparison of patient level simulation model survival outcomes after SAVR with bioprostheses and observed survival in in the Providence Health System, Portland, US by age and sex.

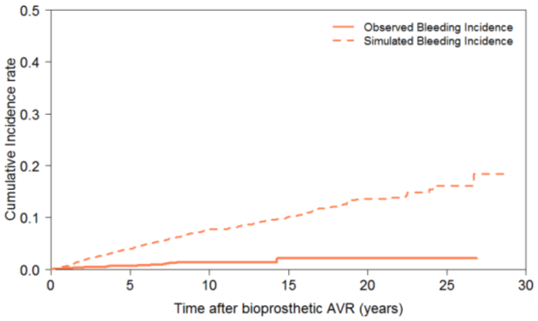

Figure S20. External validation of cumulative incidence of bleeding. Comparison of patient level simulation model cumulative incidence of bleeding after SAVR with bioprostheses and observed cumulative incidence of bleeding in in the Providence Health System, Portland, US by age and sex.

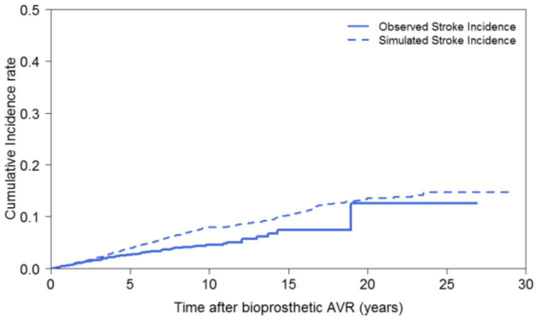

Figure S21. External validation of cumulative incidence of stroke. Comparison of patient level simulation model cumulative incidence of stroke after SAVR with bioprostheses and observed cumulative incidence of stroke in in the Providence Health System, Portland, US by age and sex.

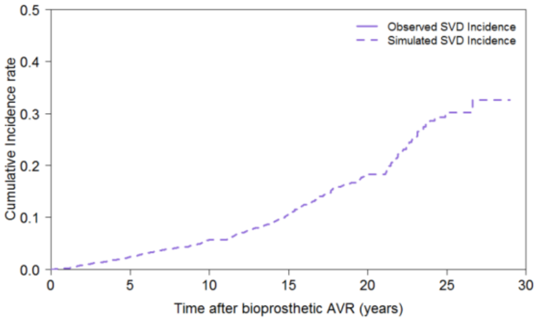

Figure S22. External validation of cumulative incidence of structural valve deterioration (SVD). Comparison of patient level simulation model cumulative incidence of SVD after SAVR with bioprostheses and observed cumulative incidence of SVD in in the Providence Health System, Portland, US by age and sex.

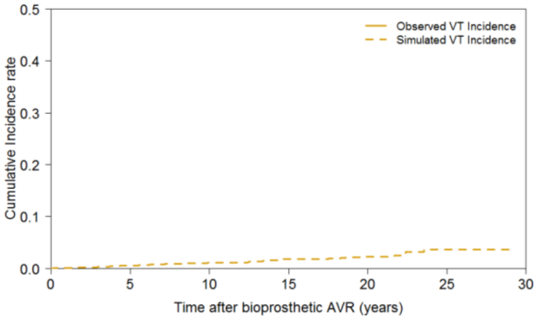

Figure S23. External validation of cumulative incidence of prosthetic valve thrombosis (VT). Comparison of patient level simulation model cumulative incidence of VT after SAVR with bioprostheses and observed cumulative incidence of VT in in the Providence Health System, Portland, US by age and sex.

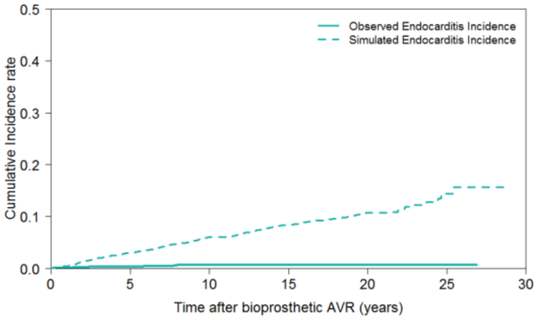

Figure S24. External validation of cumulative incidence of prosthetic valve endocarditis. Comparison of patient level simulation model cumulative incidence of endocarditis after SAVR with bioprostheses and observed cumulative incidence of endocarditis in in the Providence Health System, Portland, US by age and sex.

References

1. Huygens, S.A., et al., *How much does a heart valve implantation cost and what are the health care costs afterwards?* Open Heart, 2018. **5**(1).
2. Huygens, S.A., et al., *Conceptual model for early health technology assessment of current and novel heart valve interventions.* Open Heart, 2016. **3**(2): p. e000500.
3. Hein, R., et al., *Outcome of patients after emergency conversion from transcatheter aortic valve implantation to surgery.* EuroIntervention: journal of EuroPCR in collaboration with the Working Group on Interventional Cardiology of the European Society of Cardiology, 2013. **9**(4): p. 446-451.
4. Arsalan, M., et al., *Predictors and outcome of conversion to cardiac surgery during transcatheter aortic valve implantation.* European Journal of Cardio-Thoracic Surgery, 2018.
5. The Society of Thoracic Surgeons (STS) *STS Adult Cardiac Surgery Database Training Manual*. 2017.
6. Akins, C.W., et al., *Guidelines for Reporting Mortality and Morbidity After Cardiac Valve Interventions.* European Journal of Cardio-Thoracic Surgery, 2008. **33**(4): p. 523-528.
7. Kappetein, A.P., et al., *Updated Standardized Endpoint Definitions for Transcatheter Aortic Valve Implantation: The Valve Academic Research Consortium-2 Consensus Document†.* Journal of the American College of Cardiology, 2012. **60**(15): p. 1438-1454.
8. Huygens, S.A., et al., *Bioprosthetic aortic valve replacement in elderly patients: Meta-analysis and microsimulation.* The Journal of Thoracic and Cardiovascular Surgery, 2018.
9. Ludman, P.F., et al., *Transcatheter aortic valve implantation in the UK: temporal trends, predictors of outcome and 6 year follow up: a report from the UK TAVI Registry 2007 to 2012.* Circulation, 2015: p. CIRCULATIONAHA. 114.013947.
10. Honaker, J., G. King, and M. Blackwell, *Amelia II: A program for missing data.* Journal of statistical software, 2011. **45**(7): p. 1-47.
11. Santos, H.N.d., et al., *Predictors of stroke in patients undergoing cardiac surgery.* Brazilian Journal of Cardiovascular Surgery, 2014. **29**(2): p. 140-147.
12. Bucerius, J., et al., *Stroke after cardiac surgery: a risk factor analysis of 16,184 consecutive adult patients.* The Annals of thoracic surgery, 2003. **75**(2): p. 472-478.
13. Anyanwu, A.C., et al., *Epidemiology of stroke after cardiac surgery in the current era.* The Journal of Thoracic and Cardiovascular Surgery, 2007. **134**(5): p. 1121-1127. e1.
14. Kaireviciute, D., A. Aidietis, and G.Y.H. Lip, *Atrial fibrillation following cardiac surgery: clinical features and preventative strategies.* European heart journal, 2009. **30**(4): p. 410-425.
15. Shirzad, M., et al., *Determinants of postoperative atrial fibrillation and associated resource utilization in cardiac surgery.* Revista Española de Cardiología (English Edition), 2010. **63**(9): p. 1054-1060.
16. Mariscalco, G., et al., *Bedside tool for predicting the risk of postoperative atrial fibrillation after cardiac surgery: the POAF score.* Journal of the American Heart Association, 2014. **3**(2): p. e000752.
17. Al-Attar, N., *Postoperative Myocardial Infarction.* E-Journal of Cardiology Practice, 2011. **10**(4).
18. Sprung, M.D.P.D.J., et al., *Analysis of Risk Factors for Myocardial Infarction and Cardiac Mortality after Major Vascular Surgery.* Anesthesiology, 2000. **93**(1): p. 129-140.
19. Gargiulo, G., et al., *Transcatheter Aortic Valve Implantation Versus Surgical Aortic Valve Replacement: A Systematic Review and Meta-analysis.* Annals of Internal Medicine, 2016. **165**(5): p. 334-344.
20. Guyot, P., et al., *Enhanced secondary analysis of survival data: reconstructing the data from published Kaplan-Meier survival curves.* BMC medical research methodology, 2012. **12**(1): p. 9.
21. Franzone, A., et al., *Transcatheter aortic valve thrombosis: incidence, clinical presentation and long-term outcomes.* European Heart Journal-Cardiovascular Imaging, 2018. **19**: p. 398–404.
22. Blackstone, E.H. and J.W. Kirklin, *Death and other time-related events after valve replacement.* Circulation, 1985. **72**(4): p. 753-767.
23. Barbanti, M., et al., *5-year outcomes after transcatheter aortic valve implantation with CoreValve prosthesis.* JACC: Cardiovascular interventions, 2015. **8**(8): p. 1084-1091.
24. Mack, M.J., et al., *5-year outcomes of transcatheter aortic valve replacement or surgical aortic valve replacement for high surgical risk patients with aortic stenosis (PARTNER 1): a randomised controlled trial.* The Lancet, 2015. **385**(9986): p. 2477-2484.
25. Deeb, G.M., et al., *3-year outcomes in high-risk patients who underwent surgical or transcatheter aortic valve replacement.* Journal of the American College of Cardiology, 2016. **67**(22): p. 2565-2574.

26. Geisler, B.P., et al., *Cost-effectiveness and Projected Survival of Self-expanding Transcatheter versus Surgical Aortic Valve Replacement for High Risk Patients in a European Setting. A Dutch Analysis Based on the CoreValve High-Risk Trial.* Structural Heart, 2017(just-accepted).
27. Stevanović, J. and M.J. Postma, *Incidence description and costs of acute heart failure in the Netherlands.* Pharmacoeconomics of cardiovascular disease prevention, 2015: p. 73.
28. O'Hagan, A., M. Stevenson, and J. Madan, *Monte Carlo probabilistic sensitivity analysis for patient level simulation models: efficient estimation of mean and variance using ANOVA.* Health economics, 2007. **16**(10): p. 1009-1023.
